# Structural Investigation of Hesperetin-7-*O*-Glucoside Inclusion Complex with β-Cyclodextrin: A Spectroscopic Assessment

**DOI:** 10.3390/molecules27175395

**Published:** 2022-08-24

**Authors:** Mahendra P. Kapoor, Masamitsu Moriwaki, Katsuhiko Minoura, Derek Timm, Aya Abe, Kento Kito

**Affiliations:** 1 Nutrition Division, Taiyo Kagaku Co., Ltd., 1-3 Takaramachi, Yokkaichi 510-0844, Japan; 2 Faculty of Pharmacy, Osaka Medical and Pharmaceutical University, 4-20-1 Nasahara, Takatsuki, Osaka 569-1094, Japan; 3 Taiyo International Inc., Minneapolis, MN 55416, USA

**Keywords:** flavonoids, inclusion complex, β–cyclodextrin, hesperetin-7-*O*-glucoside, spectroscopic studies, phase–solubility measurements

## Abstract

Flavonoids are biologically active natural products of great interest for their potential applications in functional foods and pharmaceuticals. A hesperetin-7-*O*-glucoside inclusion complex with β-cyclodextrin (HEPT7G/βCD; SunActive^®^ HCD) was formulated via the controlled enzymatic hydrolysis of hesperidin with *naringinase* enzyme. The conversion rate was nearly 98%, estimated using high-performance liquid chromatography analysis. The objective of this study was to investigate the stability, solubility, and spectroscopic features of the HEPT7G/βCD inclusion complex using Fourier-transform infrared (FTIR), Raman, ultraviolet–visible absorption (UV–vis), ^1^H- and ^13^C- nuclear magnetic resonance (NMR), differential scanning calorimetry (DSC), liquid chromatography/mass spectroscopy (LC–MS), scanning electron microscopy (SEM), and powdered X-ray diffraction (PXRD) spectroscopic techniques including zeta potential, Job’s plot, and phase solubility measurements. The effects of complexation on the profiles of supramolecular interactions in analytic features, especially the chemical shifts of β-CD protons in the presence of the HEPT7G moiety, were evaluated. The stoichiometric ratio, stability, and solubility constants (binding affinity) describe the extent of complexation of a soluble complex in 1:1 stoichiometry that exhibits a greater affinity and fits better into the β-CD inner cavity. The NMR spectroscopy results identified two different configurations of the HEPT7G moiety and revealed that the HEPT7G/βCD inclusion complex has both –2S and –2R stereoisomers of hesperetin-7-*O*-glucoside possibly in the –2S/–2R epimeric ratio of 1/1.43 (i.e., –2S: 41.1% and –2R: 58.9%). The study indicated that encapsulation of the HEPT7G moiety in β-CD is complete inclusion, wherein both ends of HEPT7G are included in the β-CD inner hydrophobic cavity. The results showed that the water solubility and thermal stability of HEPT7G were apparently increased in the inclusion complex with β-CD. This could potentially lead to increased bioavailability of HEPT7G and enhanced health benefits of this flavonoid.

## 1. Introduction

Recently, there has been considerable interest in deciphering the various biological roles of flavonoids due to their higher pharmacological profiles [1,2]. Flavonoids often occur as β-glycosides of aglycones, which are widely distributed in the daily human diet via fruits, vegetables, teas, spices, herbs, and other traditional medicinal ingredients [3,4]. Hesperidin (hesperetin 7-*O*-rutinoside), a scarcely soluble monomethylated flavanone glycoside, is one of the most abundant flavonoids found in citrus peel [5,6]. The hesperetin 7-*O*-glucoside has a monosaccharide glucoside at position C7 and is the hydrolysis product of hesperidin by colonic microflora and can be readily absorbed in the small intestine (i.e., in the digestive tract) along with deglycosylated aglycone hesperetin (4′-methoxy-3′,5,7-trihydroxyflavanone), a member of the flavanone subclass of flavonoids [7,8,9].

Hesperidin, hesperetin 7-*O*-glucoside, and aglycone hesperetin have been reported to have antioxidant, anti-inflammatory, antimicrobial [10,11,12,13,14], anti-carcinogenic [15], cardio-protective [16,17,18,19], antidepressant [20], and anti-hypercholesterolemia [21,22] effects. In addition, these compounds have the capacity to reduce the risk of osteoporosis [23,24,25,26], kidney diseases [27], cytotoxicity [28], infertility, diabetes [27,29,30], and hypertension [31]. Additionally, hesperidin, including its aglycons, also exhibits significant analgesic, antiviral, antifungal, neuroprotective [32,33], and anti-cancer properties [34,35,36,37]) and is used as an auxiliary treatment associated with tiredness, allergy, muscle cramp, pain in extremities, learning/memory, and sedatives [38,39,40,41,42,43]. In addition, these flavonoids seem to be effective for treating those infected by COVID-19 in the current pandemic caused by severe acute respiratory syndrome coronavirus-2 (SARS-CoV-2) [44].

The use of hesperidin as a natural herbal supplement is greatly limited by its very poor aqueous solubility (i.e., less than 20 ppm) [45] and low bioavailability (< 2 μmol/L) [46] in human subjects, which limit its therapeutic potential. The low bioavailability of hesperidin has been attributed to the rutinoside moiety attached to the flavonoid [47,48], and due to the delayed action of the intestinal microbiota needed to release the rutinoside of hesperidin prior to the absorption of its aglycone hesperetin. Nielsen et al., demonstrated that the removal of the rhamnose sugar moiety from hesperidin by enzyme improved the bioavailability of the aglycone hesperetin threefold in human subjects [49]. In line with the aforementioned concept, Furtado et al., studied the enzymatic production of hesperetin from hesperidin with soluble as well as immobilized hesperidinase enzymes targeting improved bioavailability [50]. A single-blind, randomized, two-phase crossover clinical trial involving the direct in vivo perfusion of the jejunum and oral ingestion of an equimolar amount of purified hesperidin and hesperetin-7-*O*-glucoside was carried out in healthy subjects. The results showed a better absorption of hesperetin-7-*O*-glucoside compared with hesperidin in the small and large intestine, confirming the role of colonic microbiota [51]. Wan et al. constructed an immobilized enzyme catalysis platform using immobilized rhamnosidase on an Fe_3_O_4_@graphene oxide, for preparing a hesperidin complex as a soluble substrate, and ammonium hydroxide as a ligand dissociation agent to obtain hesperetin-7-*O*-glucoside [52].

Furthermore, several approaches to improve the solubility of the flavonoids, including the formation of host–guest inclusion complexes, have been attempted. Cao et al. described the formulation of a hesperidin and chitooligosaccharides complex for improved antioxidant properties than hesperidin alone [53]. Current strategies to circumvent the low solubility and stability problems enabling more efficient bioavailability of flavonoids is to develop a suitable method using cyclodextrins (CDs) as entrapping vehicles. Cyclodextrins already have a long history of diverse applications in the food, cosmetic, textile, chemical, environmental, agriculture, biotech, and pharmaceutical industries for their solubility, stability, safety, bioavailability, and as an encapsulating agent or carrier of the functional ingredient of potential health interest [54]. The advantage of cyclodextrin is the simplicity of the chemical modification due to the presence of hydroxyl groups on the well-defined hydrophobic cone-like central inner cavity (non-polar) and outer hydrophilic rim surface (polar) of the cyclodextrins. Additionally, the complexation with cyclodextrins does not alter the chemical structure of flavonoids because they are linked mainly by van der Waals forces and hydrophobic interactions [55,56].

β-Cyclodextrin is a macrocyclic oligosaccharide comprising seven glucopyranose units bound by α-1, 4 glucosidic linkages forming a fairly rigid truncated conical structure [54,57]. Despite the low solubility of β-cyclodextrin compared with α and γ-cyclodextrins, it is preferred and more widely used as complexation because of its perfect cavity size, stable rigid structure due to the higher stability of intramolecular hydrogen bonds, and low production cost [58,59]. Supramolecular inclusion complexes of hesperidin with cyclodextrins have also been studied in extensive detail [54,60]. Corciova et al. [12] demonstrated enhanced antibacterial and antioxidant properties and improved dissolution profiles of hesperidin-β-cyclodextrin inclusion complexes prepared by different techniques (such as kneading, co-evaporation, and lyophilization). Ficarra et al. [61] investigated hesperetin and hesperidin complexed with β-cyclodextrin, and improvements in chemical stability and solubility were demonstrated. Tommasini et al. [62] reported the inclusion complex of hesperetin and hesperidin with 2-hydroxypropyl-β-cyclodextrin (HP-β-CD) of 1:1 stoichiometry, wherein better complexation was observed by hesperetin and HP-β-CD compared with that assessed for hesperidin. Corciova et al. [63] also reported that complexing hesperidin with HP-β-CD resulted in improved dissolution as well as antioxidant and antibacterial properties. Similarly, complexing hesperetin with β-CD and HP-β-CD enhances the dissolution rate and improves solubility, along with anti-inflammatory and cytotoxicity effects on cancer cell lines [64,65,66].

In the present study, we report the formulation of a proprietary water-soluble hesperetin-7-*O*-glucoside inclusion complex with β-cyclodextrin (HEPT7G/βCD; SunActive^®^ HCD). We aim to investigate the potential stability, solubility, and spectroscopic features of the HEPT7G/βCD inclusion complex using Fourier-transform infrared (FTIR), Raman, ultraviolet–visible absorption (UV–vis), ^1^H- and ^13^C-nuclear magnetic resonance (NMR), differential scanning calorimetry (DSC), zeta potential, liquid chromatography/mass spectroscopy (LC-MS), scanning electron microscopy (SEM), and powdered X-ray diffraction (PXRD) spectroscopic techniques, including Job’s plot and phase solubility measurements. We were particularly interested in exploring the details of supramolecular interactions and the consequences of the complexation of the HEPT7G moiety with a β-CD molecule on improved structural stability, physicochemical properties, and enhanced water solubility for the enhanced bioavailability of HEPT7G/βCD inclusion complex.

## 2. Materials and Methods

### 2.1. Materials

β-Cyclodextrin (β-CD; purity > 98%) was purchased from CycloChem Co., Ltd., Kyoto, Japan (a product of Wacker Chemie AG, Munich, Germany). Commercial hesperidin (purity > 97%) was supplied by Hamari Chemicals, Ltd., Osaka, Japan. HPLC-grade hesperidin reference standard (purity > 97.0%; molecular weight, 610.56 Da) was purchased from Fuji Film Wako Pure Chemical Corporation, Osaka, Japan. HPLC-grade regent methanol, phosphoric acid, and formic acid were purchased from Nacalai Tesque, Inc., Tokyo, Japan. HPLC reagent acetonitrile was supplied by Kanto Chemical Co. Inc., Tokyo, Japan. Reference standard hesperetin-7-*O*-glucoside (purity > 96%; molecular weight 464.38 Da) was purchased from Sigma-Aldrich, St. Louis, MO, USA, and the Fuji Film Wako Pure Chemical Corporation. *Naringinase* enzyme was obtained from Amano Enzyme Inc., Nagoya, Japan. All commercially available reagents used for analyses were of very high purity. The solvents used in the spectrophotometric analysis were of spectroscopic reagent grade, and analytical-reagent-grade chemicals were used. The calculated and exactly weighed amounts (1:1 molar ratio) of desiccator dried pure HEPT7G (standard) and β-CD were pulverized in a ceramic mortar and mixed carefully to obtain an HEPT7G+βCD physical mixture.

### 2.2. Hesperetin-7-O-Glucoside Inclusion Complex with β-Cyclodextrin

**Figure 1:**
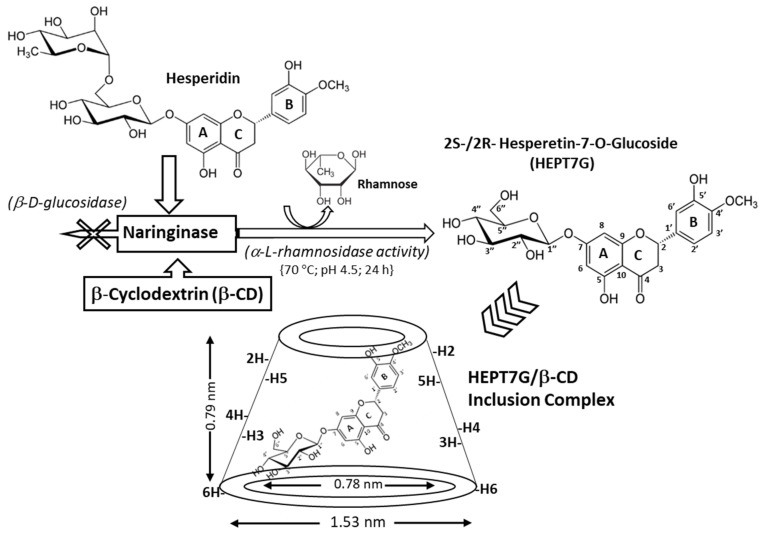
A schematic illustration depicting the formulation of the proprietary hesperetin-7-*O*-glucoside/β cyclodextrin inclusion complex (HEPT7G/βCD) via the enzymatic hydrolysis of hesperidin with naringinase in the presence of β-CD, wherein the hesperetin-7-*O*-glucoside moiety completely entered into the truncated cone structure cavity of β-CD for HEPT7G/βCD inclusion complex formation.

The patented composition containing the Hesperetin-7-*O*-glucoside inclusion complex with β-cyclodextrin (HEPT7G/βCD), wherein hesperetin glycosides were included in β-cyclodextrin through controlled enzymatic hydrolysis of hesperidin (HEPD; derived from a *citrus Aurantium extract* containing 90.3% hesperidin) with *naringinase* as an enzyme [67] [SunActive^®^ HCD; HEPT7G content: 14.5% (*w*/*w*); β-CD content: 57.5% (*w*/*w*); manufacturer: Taiyo Kagaku Co., Ltd., Japan]. In large-scale production of this proprietary formulation, the hesperidin and β-cyclodextrin in water were mixed in a tank at 70 °C and pH was adjusted to 4.5, followed by *naringinase* digestion performed for 24 h. The conversion rate was determined to be nearly 98%, estimated using high-performance liquid chromatography (HPLC) analysis. Subsequently, the product solution was filtered, sterilized with microfiltration, and then dried in a spray dryer. The percentage of HEPT7G content linked with the HEPT7G/βCD inclusion complex was determined using spectrometric analysis, and finally, the HEPT7G/βCD inclusion complex was stored at room temperature in ambient relative humidity. The production of different lots of product confirmed that the formation of the HEPT7G/βCD inclusion complex was a repeatable process. The chemical structure of the HEPT7G/βCD inclusion complex and molecular hesperetin-7-*O*-glucoside are displayed in Figure 1.

### 2.3. Characterization Methods of Hesperetin-7-O-Glucoside/β-Cyclodextrin Inclusion Complex

#### 2.3.1. Vibrational Spectroscopy

Fourier-transform infrared (FTIR) and Raman variants of vibrational spectroscopic technique were used as a complement to each other for the identification of functional groups and bond characterization of HEPT7G/βCD inclusion complex to achieve a broader set of research goals.

FTIR spectra of the potassium bromide mixtures of the samples at a ratio of 1:100 were obtained by gently mixing in an agate mortar, as KBr pellets (compressed disk prepared using a hydraulic press) with the β-CD, HEPT7G (standard), HEPT7G+βCD physical mixture, and HEPT7G/βCD inclusion complex were collected on a diffuse reflectance Fourier-transform infrared spectrophotometer instrument (Shimadzu Corp., Kyoto, Japan) to analyze their chemical properties. Spectral scanning data were acquired within the wavenumber range between 4000 and 400 cm^−1^ using the following conditions: operating mode: transmission percentage; wavenumber resolution: 2 cm^−1^; cumulative scans: 64 times at room temperature to obtain an appropriate signal-to-noise ratio for Fourier self-deconvolution and band fitting. The Happ–Genzel apodization function, which gives the precise mathematical effect on the computed infrared spectrum without large artifacts, was applied.

The FT–Raman spectra were recorded on a Raman spectrometer analyzer (Renishaw inVia, Gloucestershire, United Kingdom) using a diode pump solid-state laser with an excitation wavelength (λ_exc_) of 785 nm. The laser power of the source was maintained at 25 mW between 10 s and 120 s for all samples {i.e., β-CD, HEPT7G (standard), HEPT7G+βCD physical mixture, and HEPT7G/βCD inclusion complex}. All FT–Raman spectral data were acquired between 5000 and 100 cm^−1^ using the wavenumber resolution 1 cm^−1^ by irradiating the samples with laser light. The experimental FTIR and FT–Raman spectra of all samples with their scaled frequencies were compared.

#### 2.3.2. Nuclear Magnetic Resonance Spectroscopy (NMR Analysis)

^1^H- and ^13^C-NMR experiments are useful techniques to investigate the possible inclusion mode and relative orientation of flavonoid guest molecules inside the hydrophobic cavity of cyclodextrin host molecules. We performed ^1^H- and ^13^C-NMR spectroscopic analysis of the HEPT7G/βCD inclusion complex, which was very helpful in elucidating the molecular conformation of the inclusion complex between β-cyclodextrin with HEPT7G. ^1^H- and ^13^C-NMR spectra were recorded on an Agilent Technologies NMR system 600 DD_2_ spectrometer operating at 600 MHz at 298 K in deuterium oxide (D_2_O; purity 99.9 atom% D) as a solvent. The chemical shift (δ) values are reported in ppm using 3-(trimethylsilyl) propionic-2,2,3,3,-d4 acid (TSP-D4; 0 ppm) as the internal standard. Where necessary, a small portion of DMSO-d_4_ was used as an added solvent to dissolve the sample crystals completely. All spectra were acquired under standard conditions.

Two-dimensional total correlation spectroscopy (2D-TOCSY) and NMR spectra were acquired using standard pulse sequences from the Agilent library. ^1^H homonuclear two-dimensional nuclear overhauser effect spectroscopy (2D-NOESY) and rotating overhauser effect spectroscopy (2D-ROESY) spectra were also acquired for the inclusion complex of β-cyclodextrin with HEPT7G to confirm the additional assessments of all hydrogens. All spectra were acquired at 298 K with pre-saturation of the residual water resonance. The experimental conditions were as follows: software: VNMRJ rev. 3.2A; spectral width, 4926.6 Hz; acquisition time, 0.15 s; pulse width, 9.3 Hz; data points, 1388; relaxation delay between successive pulse cycle, 1.3 s, and 32 scans. The data were processed with an exponential multiplication window function. To determine the spatial arrangement within the cavity of β-cyclodextrin with HEPT7G in the HEPT7G/βCD inclusion complex, changes in the chemical shift (δ, ppm) of the principal signals in the spectra of β-CD, HEPT7G (standard), and the HEPT7G+βCD physical mixture were evaluated.

#### 2.3.3. Thermal Analysis

Differential scanning calorimetry (DSC) curves were performed using a TA instruments differential scanning calorimeter model DSC 250, equipped with Tzero technology and autosampler features including a heat flux system (temperature range: −180 to 725 °C; DSC range: ±500 mW; enthalpy precision: ±0.08%; temperature accuracy: ±0.05 °C). The instrument was pre-calibrated for the temperature and sensitivity with indium, according to the standard procedure. The measurements were made in duplicates for the HEPT7G/βCD inclusion complex and HEPT7G+βCD physical mixture. About 2.0 mg of each sample was placed and heated in crimped aluminum pans with a small hole in the lid at a constant heating rate of 10 °C/min from room temperature to 250 °C with dynamic nitrogen purging as an inert atmosphere with a flow rate of 50 mL/min. For increased accuracy, intuitive software with the auto analysis function of thermal analysis data was applied.

#### 2.3.4. UV–Visible Spectroscopy

The UV–visible absorption spectra of the pure reference standard HEPT7G, HEPT7G/βCD inclusion complex, and HEPT7G+βCD physical mixture were obtained with a Cary 60 UV–Vis (ver. 2.0; scan software ver. 5.0 v) double beam spectrophotometer (Agilent Technologies Japan, Ltd., Tokyo, Japan) fitted with fiber optic probes to quantify the amount of HEPT7G included in the materials without baseline correction. The concentration was calculated by the interpolation of calibration curves previously obtained from the HEPT7G (standard) and β-CD. Quartz cells with a 10 mm path length were used in the UV spectral scan range of 200–500 nm with a scanning speed of 30 nm/min and a fixed slit width of the data interval of 1 nm.

#### 2.3.5. X-Ray Powder Diffraction (PXRD)

X-ray diffraction patterns of the HEPT7G/βCD inclusion complex were analyzed and compared with the spectra of β-CD and the HEPT7G+βCD physical mixture. The PXRD spectra were recorded in ambient temperature conditions using a Rigaku RINT X-ray diffractometer model 2200 V (Rigaku Corporation, Tokyo, Japan). Each measurement was repeated at least twice. The X-ray source was Ni-filtered CuKα radiation (λ = 1.5418 Å). The range (2θ) of scans interval was 5° ≤ 2θ ≥ 40° with a step size of 0.05° at an angular scan speed of 1° 2θ min^−1^, at 300 K. Other scanning conditions were as follows: tube voltage: 40 kV; target: Cu; time constant: 4 s; current: 30 mA; slit width: 2/3° (divergence), and 1/2° (scattering).

#### 2.3.6. Liquid Chromatography–Mass Spectrometry (LC-MS/MS)

The formation of HEPT7G was further confirmed using a high-resolution LC-MS/MS analyzer, a fully integrated triple-quadrupole mass spectrometer (LC: 20A series, Shimadzu Corporation, Kyoto, Japan; MS: API 3200 equipped with Analyst^®^1.6.2 Applied Biosystem/MDS SCIEX software (AB Sciex Pte. Ltd. Singapore). The parameters for the LC-MS/MS analysis were as follows: column: Mightlysil RP-18GP; particle size used: 3 μm; column size: 3.0 mm (φ) × 150 mm (length); column temperature: 40 °C; mobile phase composition: (A) 0.1% formic acid water solution, and (B) acetonitrile with 0.1% formic acid; flow rate: 0.3 mL/min; injection volume: 5 μL; ionization mode: ESI (negative; −4500 V). Analysis was performed under gradient conditions (time = 0 min 10% solvent B; time = 10 min 95% solvent B; time = 12 min 95% solvent B; time = 12.1 min 10% solvent B; time = 15 min 10% solvent B). For all the samples, duplicate injections were performed with the described LC-MS/MS analytical method using the auto-sampler conditioned at 4 °C.

#### 2.3.7. Scanning Electron Microphotographs

The surface morphologies of β-CD, hesperidin, HEPT7G (standard), the HEPT7G+βCD physical mixture, and the HEPT7G/βCD inclusion complex were observed using a Hitachi Miniscope 7247 scanning electron microscope. Before the measurement, the dry powder samples were mounted and evenly spread onto carbon-coated tabs using double adhesive carbon-coated tape adhered to stainless steel stubs. Photomicrographs were scanned at an excitation voltage of 10 kV under vacuum conditions and various magnifications between ×100 and ×1000 with direct data capture of the images by computer. No thin layer of gold sputtering was applied to make an electrically conductive sample surface because high-resolution images were achieved at low accelerating voltages through charge compensation without any coating.

#### 2.3.8. Zeta Potential (ζ)

The Zeta (ζ) potential was determined with a ZetaSizer ZS90 analyzer (Malvern Instruments, UK) equipped with a photodiode detector (4 mW He-Ne-Laser with a wavelength 633 nm) using the micro-electrophoresis method and processing of the images with NanoSight v. 3.1 software. In aqueous solution, the zeta potential depends on the pH value; therefore, the zeta potential was estimated from the average of five measurements of 60 s at pH 7.0, at ambient temperature (nearly 25 °C), on the diluted solution of pure HEPT7G (standard) and HEPT7G/βCD inclusion complex particles to confirm the complex formation.

#### 2.3.9. Solubility Determination and Phase–Solubility of HEPT7G/β CD Inclusion Complex

The solubility of HEPT7G (standard), HEPT7G+βCD physical mixture, and the HEPT7G/βCD inclusion complex was determined by ultraviolet spectroscopy (Agilent Technologies Japan, Ltd., Tokyo, Japan). For solubility estimations of the HEPT7G/βCD inclusion complex, the reaction-terminated liquid mixture was allowed to stand at room temperature, then filtered and lyophilized. In a 100 mL beaker, the dried product sample was dissolved in 50 mL of distilled water (specific conductivity of 1 × 10^−6^ S cm^−1^) at 50 °C with continuous stirring until the saturation equilibrium was reached (i.e., product no longer dissolved and precipitated out). After cooling the saturated solution under ambient conditions and standing at room temperature (25 °C), 1 mL of the supernatant was then filtered and stored for absorption metric analysis. HEPT7G (standard) and the HEPT7G+β CD physical mixture were dispersed in distilled water in a 10 mL screw-capped glass bottle (1.0% solution equivalence). The solutions were continuously shaken at ambient conditions for 24 h to ensure that equilibrium was attained. The equilibrated sample solutions were centrifuged at 5000× *g* for 10 min and filtered with Millipore filtration to remove sediments of undissolved fractions. The supernatant collected was used to estimate HEPT7G solubility in the samples, and further diluted to measure the absorbance between 284 to 286 nm. All measurements were conducted in triplicate. The HEPT7G standard solution was prepared in a concentration of 1.0% solution equivalence in DMSO/water and its successive dilution was prepared and used to plot a calibration curve to estimate the HEPT7G solubility in water from HEPT7G (standard), the HEPT7G+βCD physical mixture, and the HEPT7G/βCD inclusion complex.

For the phase–solubility measurements, the rate profile of HEPT7G inclusion into the proprietary HEPT7G/βCD molecular inclusion complex was determined according to the production method, wherein controlled enzymatic cleavage reaction selectively hydrolyzed the non-reducing terminal rhamnose from hesperidin. The reaction-terminated liquid mixtures of varied compositions were allowed to stand at room temperature, then filtered and lyophilized to obtain dried products according to the method stated above. After termination of the reaction, the supernatant samples (filtered through a 0.45 μm pore cellulose acetate membrane, mm) were collected with varied HEPT7G to β-CD molar ratios, having rhamnose residue removed by dialysis of the filtrate at ambient temperature, were analyzed for hesperetin-7-*O*-glucoside concentration using HPLC analysis (Shimadzu Corp., Kyoto, Japan) as reported elsewhere [67].

The filtered sample solutions were further adequately diluted to quantify the HEPT7G by spectrophotometry assay measuring the absorbance at *λ*_max_ @282 to 285 nm. The results were subjected to phase–solubility measurements. A phase–solubility diagram was obtained by plotting the molar concentration of solubilized HEPT7G as a function of β-CD molar concentrations. The apparent solubility in terms of binding or association constant (K_1:1_, M^−1^) of the proprietary HEPT7G/βCD molecular complex formation was estimated from the slope of the phase–solubility diagram according to the following equation:

Ks (M^−1^) = slope/S_0_ (1 − slope);

where S_0_ is the intrinsic HEPT7G solubility at room temperature in the absence of β-CD.

Furthermore, the Job’s plot method is used to estimate the stoichiometry in cyclodextrin complexation, whereas some reports have replaced the Job’s plot with a phase–solubility study. In the support of our phase–solubility measurement study, we have also estimated the stoichiometry of the HEPT7G/βCD inclusion complex using Job’s plot method. A set of solutions for the HEPT7G moiety and β-CD was prepared with varying amounts of molar fraction of the HEPT7G in the range (R) 0 to 1, and UV–Vis absorbance was estimated. The Job’s plot was obtained by plotting Δ _abs_ × R _concentration_ against R _concentration_, wherein Δ _abs_ is the difference in absorbance of the HEPT7G moiety without and with β-CD, and R _concentration_ = [HEPT7G]/{[HEPT7G] + [β-CD]}.

#### 2.3.10. High-Performance Liquid Chromatography (HPLC)

The HPLC analysis was performed using the Shimadzu HPLC system (Nexera XR; Shimadzu Corp., Kyoto, Japan). The parameters for the HPLC analysis were as follows: column: CAPCELL PAK C18 (Shiseido, Japan); size: 4.6 mm (φ) × 250 mm, detector: ultraviolet (UV) detection at wavelength 280–285 nm; mobile phase: 40% (*v*/*v*) acetonitrile/0.1% aqueous phosphoric acid solution; flow rate: 0.4 mL/min; column temperature: 70 °C. The autosampler was used and the injection volume (5–20 μL) differed depending on the assay. Standard hesperidin and HEPT7G were used to validate the HPLC method. The analysis was performed in duplicate, as reported elsewhere (Moriwaki, Kumoi, and Ozeki, 2019).

## 3. Results and Discussion

Several reports suggest improvements in the flavonoid solubility by forming inclusion complexes with cyclodextrins [68,69,70]. The proprietary inclusion complex of hesperetin-7-*O*-glucoside with β-CD (HEPT7G/βCD; SunActive^®^ HCD) is the patented formulation prepared via the controlled enzymatic hydrolysis of hesperidin in the presence of β-CD. The objective of the present study is to report the characteristic evaluation of the inclusion complexation process of hesperetin-7-*O*-glucoside with β-CD with improved solubility and the potentially enhanced bioavailability of hesperetin aglycone metabolites after oral administration. The truncated cone-shaped doughnut-structured hydrophobic cavity of β-CD has the capability to clathrate the HEPT7G as a guest molecule to form a stable inclusion complex through host–guest complexation in an aqueous solution. The HEPT7G molecule formed by the selective enzymatic hydrolysis of hesperidin using naringinase enzyme enters the β-CD cavity after the high-energy water molecule is released from the cavity of β-CD, establishing a thermodynamic equilibrium through the homogenous distribution of a polar–apolar association under a controlled reaction environment, wherein the reaction temperature influences the selectivity of the energetically favored binding or interaction between the semi-polar interior cavity of β-CD and the HEPT7G along with other factors such as enthalpy, entropy, and Gibbs free energy of the complexation system. The formation of the inclusion complex of HEPT7G with β-CD was a spontaneous and repeatable process.

The chemical structure of the proprietary inclusion complex of HEPT7G with β-CD is displayed in Figure 1. Several analytical techniques were used to evaluate the immobilized matrix properties, structural configuration, and physical state of the powdered HEPT7G inclusion complex with β-CD, and the results indicated the best interpretations in terms of structural confirmation of the complexation process.

### 3.1. Vibrational Spectral Change Analysis of Inclusion Complex Formation

**Figure 2:**
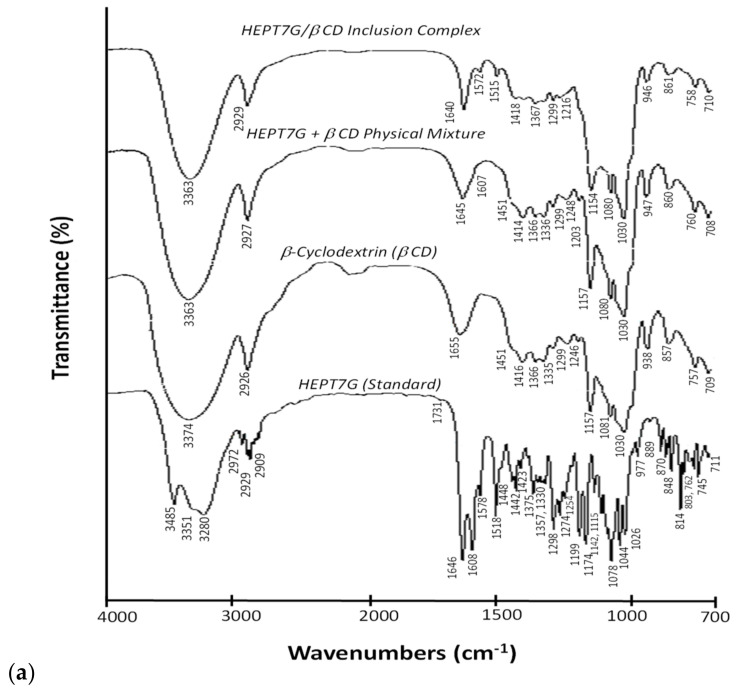

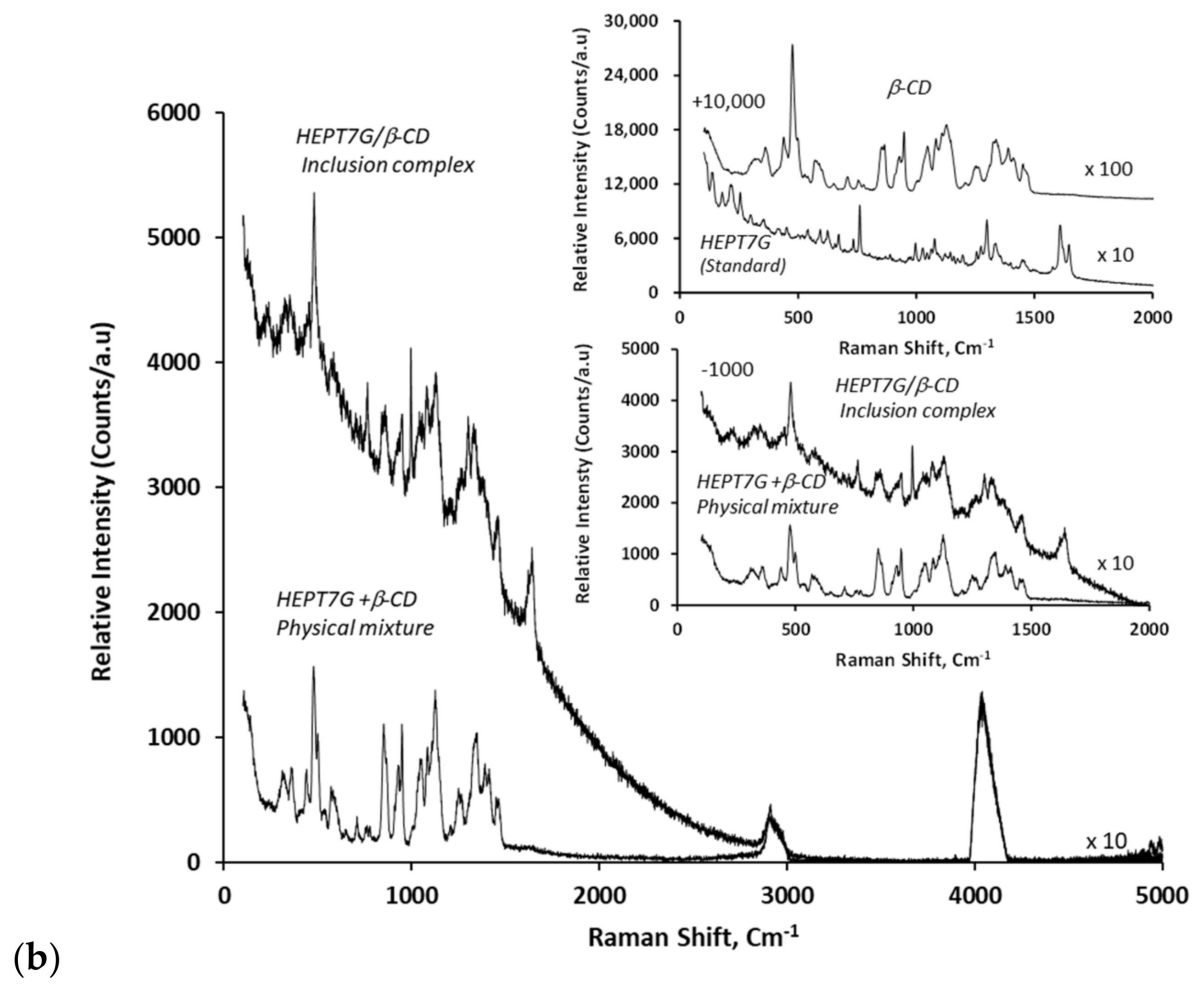
(**a**) FTIR transmittance spectra for β–CD, pure HEPT7G (standard), HEPT7G+βCD physical mixture, and HEPT7G/βCD inclusion complex, and (**b**) FT–Raman scattering spectra of the HEPT7G+βCD physical mixture and HEPT7G/βCD inclusion complex. Raman scattering spectra for β-CD, pure HEPT7G (standard), and HEPT7G+βCD physical mixture are compared with HEPT7G/βCD inclusion complex in the 0 to 2000 cm^−1^ interval (Inset).

FTIR spectroscopy was applied to ascertain the formulation of the inclusion molecular complex of hesperetin-7-*O*-glucoside with β-CD in the powdered state. The spectra of the HEPT7G (standard), β-CD, HEPT7G/βCD inclusion complex, and HEPT7G+βCD physical mixture are depicted in Figure 2a. The analysis of the FTIR spectra was carried out at 4000–400 cm^−1^. In the FTIR spectra of HEPT7G (standard), the prominent absorption bands indicate the crystalline profile of the molecule. The main characteristic vibrational modes of the HEPT7G (standard) molecule can be assigned, as follows: phenolic v (–OH) symmetric and asymmetric bands distinguished by three maxima at 3485, 3351, and 3280 cm^−1^ associated with the valence vibration of an intermolecular bond of primary hydroxyls (i.e., band at 3485 cm^−1^), and intramolecular bonds of secondary hydroxyl of the glucopyranosyl units (i.e., bands at 3351 and 3280 cm^−1^); v (–CH) stretching absorption bands at 2972, 2929, and 2902 cm^−1^; stretching absorption band at 1646 and 1608 cm^−1^ associated with v (–C=C) and v (–C=O) carbonyl conjugation and the stretching of aromatic benzene rings, respectively; v (–C=C) vibrations corresponding to the aromatic rings appearing near the 1400–1300 cm^−1^ absorption region; predominant vibrations between 1300 and 700 cm^−1^ corresponding to stretching and bending frequencies to aromatic rings; and the methoxylic band at 1274 cm^−1^. The modifications observed in characteristic spectral absorption bands in the FTIR spectra confirmed the formation of the HEPT7G/βCD inclusion complex. The most intensive changes in the spectral features, such as wavenumber shifts, modified intensities, the disappearance of the characteristic bands, and the existence of new species with different spectroscopic features, were confirmed in spectral ranges associated with concerned bending and stretching vibration regions.

The β-CD spectrum demonstrates the presence of the most characteristic wide absorption band centered at about 3374 cm^−^^1^, a v(–OH) phenolic hydroxyl stretching vibration mode of the β-CD bridged system, along with a sharp symmetric and asymmetric stretching band v(–CH) at 2926 cm^−^^1^ (–CH aliphatic stretching vibration). A similar broad vibrational band appeared in the spectrum of the HEPT7G/βCD inclusion complex, but was slightly narrowed with the center shifted (−11 cm^−1^) to a lower wave number at 3363 cm^−^^1^ due to hydrogen bonding between the narrow-side hydroxyl groups of the β-CD cavity that could have collapsed and eventually disappeared after the formation of inclusion complex. Additionally, their varied intensities could be attributable to alterations in the hydrogen bonding of HEPT7G segments in the inner semi-polar cavity of β-CD. However, no characteristic shifts in the location or intensity of the stretching band at 2927 cm^−1^ were noticed. An additional peak observed at 1655 cm^−1^ in the β-CD spectrum was associated with the H-O-H deformation band of adsorbed water present in the β-CD cavity, referred to as δ (-OH) groups of glucopyranosyl units (see Figure 2a). This peak disappeared in the spectra of the HEPT7G/βCD inclusion complex due to the release of high-energy water molecules during the formation of the inclusion complex of HEPT7G with β-CD, further confirming the formation of the HEPT7G/βCD inclusion complex. In addition, a small shoulder at 1731 cm^−1^ which appeared in the HEPT7G (standard) spectrum also disappeared from the spectrum of the HEPT7G/βCD inclusion complex, which indicates the successful formation of the inclusion complex.

The stretching absorption bands at 1645 and 1607 cm^−1^ of the HEPT7G moiety, associated with v (–C=C) and v (–C=O) carbonyl conjugation and stretching of C=C valence vibration in the aromatic benzene rings, respectively, were slightly shifted and broadened with reduced intensity in the HEPT7G/βCD inclusion complex. Despite all vibrational bands associated with β-CD being observed in the spectrum of the HEPT7G/βCD inclusion complex, the characteristic spectrum of HEPT7G (standard) was substantially different from that of HEPT7G/βCD inclusion complex in the 1600–1200 cm^−1^ absorption region. The characteristic absorption band of HEPT7G (standard) at 1442 cm^−1^ attributed to the distribution of aromatic rings of the HEPT7G moiety disappeared in the HEPT7G/βCD inclusion complex, generalizing that the aromatic rings of HEPT7G/βCD inclusion complex within the β-CD cavity were composed either of van der Waals forces and/or regulated hydrophobic interactions. Additionally, the methoxylic band of HEPT7G (standard) disappeared in the FTIR spectrum of the HEPT7G/βCD inclusion complex. The strong bands corresponding to the stretching vibration modes of the v (–C=C), v (–C=O), and wagging vibration mode of δ (C-H) bonds in the HEPT7G (standard) and HEPT7G/βCD inclusion complex were also observed in the region 1200–700 cm^−1^. After complexation, the changes were visible for some predominant bands. The intensities of the band related to the carbonylic group decreased. The absorption bands at 1080 and 1030 cm^−1^ are associated with the overtone v (C-H) stretching mode, and that at 1030 cm^−1^ corresponds to v (–C-H), v (–C=O) stretching modes. The inclusion of the HEPT7G moiety in the β-CD cavity did not affect the strong vibrational band at 1157 cm^−1^ attributed to v (C-O-C) vibration. Other wagging vibration bands with varied intensities and wavenumber shifts in the fingerprint region were still visible, including the absorption peaks at 947 and 860 cm^−1^ corresponding to the presence of glucopyranosyl units. This suggests that the A, B, and C rings of the HEPT7G moiety are located in the proximity of the ring oxygen atom of the β-CD cavity. Furthermore, the similarities of the HEPT7G/βCD inclusion complex and physical mixture HEPT7G+βCD spectra might be associated with the force applied to obtain the physical mixture that promotes non-covalent interactions between species and could lead to partially identical features of HEPT7G/βCD inclusion complex. The results also suggest that the interaction between β-CD and the HEPT7G moiety is largely by the aromatic ring structure. Overall, the results demonstrated that the HEPT7G moiety was included in the β-CD cavity, because a majority of the corresponding absorption bands either completely disappeared or shifted to higher or lower wavenumbers. The relative changes are listed in Table 1 along with brief descriptions. The FTIR spectrum of the HEPT7G/βCD inclusion complex presented in Figure 2a depicts a change in its profile when compared with the spectrum of the HEPT7G (standard) molecule and suggests the incorporation of the HEPT7G moiety into the β-CD inner cavity.

The Raman vibrational spectra of the HEPT7G (standard), β-CD, and HEPT7G/βCD inclusion complex along with a physical mixture of HEPT7G+βCD in a KBr pellet are displayed in Figure 2b. The C=H stretching modes appeared with Raman intensity and are generally highly polarized. Typically, Raman scattering in the 1700–1200 cm^−1^ and 1200–300 cm^−1^ vibrational regions was evaluated. The spectrum of the HEPT7G/βCD inclusion complex consists of the bands with varying intensities and shapes observed at 1641 and 1607 cm^−1^, the stretching vibration corresponding to aromatic ring carbonyls v (–C=O) and v (–C=C) conjugation in stretching mode. The similarity of the inclusion complex and physical mixture of HEPT7G with β-CD also observed in the Raman spectra except a small shoulder appeared at 1646 cm^−1^ further supports the findings of FTIR results. The frequencies of the predominant Raman bands are summarized in Table 1, along with a brief description of their assignments. In the Raman spectrum of the HEPT7G (standard) molecule, the strong bands were located at 1646, 1609 (stretching C=O and C=C bands), and 1576 cm^−1^ coupled with a small peak at 1485 cm^−1^ was associated with stretching vibration of the conjugated aromatic ring structure (A and C rings). Additionally, the strong bands observed in the Raman scattering spectra in the 1500–1300 cm^−1^ region with different intensities and shifts suggest the involvement of the C=O, carbonyl C-O bonds, as well as C=C bonds in the substituent in the formation of hydrogen bonding for the HEPT7G moiety in complex with β-CD. The assignments are further supported by Raman scattering intensities in the 1300–1100 cm^−1^ region, which corresponds to the bending vibration of the C-O-H heterocyclic bonds and C-C-H bond in aromatic rings in the ring structure. The other most characteristic differences of bands associated with hydrogen bonds that appeared in the HEPT7G/βCD inclusion complex were located at the 1000–700 cm^−1^ Raman frequency region. It was possible to identify the appearance of hydrogen bonds in the above range, as an effect of a decrease in the intensity of the wagging- and twisting-type vibrations modes of hydroxyl groups directly linked to sugar moieties. Due to the significant susceptibility of the carbonyl group to hydrogen bonding, the broadening and shifting of the bands in the 1000–700 cm^−1^ range also support the formation of the HEPT7G/βCD inclusion complex. The spectrum of physically mixed HEPT7G+βCD was nearly the arranged sum of the HEPT7G molecule and β-CD, but exhibited reduced peak intensities with partial peak shifts, confirming the inadequate complex formation in the physically mixed HEPT7G+βCD sample. Therefore, the Raman scattering spectra results preferentially support the FTIR spectroscopy results demonstrating that the HEPT7G moiety was successfully contained in the inner cavity of β-CD. The differences in the vibrational intensities of the predominant bonds are attributable to the restricted vibration of the aromatic ring structure of HEPT7G moiety in the β-CD cavity.

### 3.2. Inclusion Complex Formation Study by Nuclear Magnetic Resonance Analysis

NMR spectroscopy has been recognized as the most powerful analytical tool for the structural elucidation of flavonoids developed thus far [71]. The NMR studies provide useful evidence about the spatial proximity between host and guest intermolecular interactions. The formation of complexes leads to chemical shifts that provide valuable information and unambiguous evidence on the nature of the complexation. We performed ^1^H- and ^13^C-NMR spectroscopic studies to investigate the possible inclusion mode and the relative orientation of the hesperetin-7-*O*-glucoside (guest) molecule inside the hydrophobic cavity of the β-CD (host) molecule by observation of the qualitative differences between in the chemical shifts (Δδ) associated with the HEPT7G/βCD inclusion complex and free HEPT7G molecule, using the following equation:

Chemical shifts (Δδ) = δ (HEPT7G/βCD inclusion complex) − δ (free HEPT7G moiety)

Furthermore, the assignment of NMR frequencies was supported by the coupling constants, signal splitting, and other related information due to the absence of any direct chemical bonding between the HEPT7G moiety and β-CD molecule in the HEPT7G/βCD inclusion complex.

**Figure 3:**
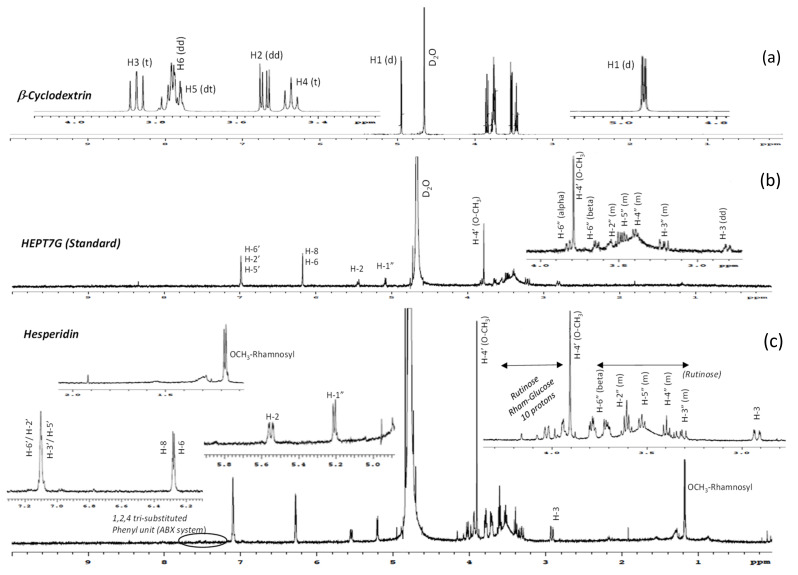
^1^H-NMR spectra of (**a**) β–CD, (**b**) pure HEPT7G (standard), and (**c**) hesperidin (raw material). The chemical shift ranges of well-resolved resonance peaks of associated protons of main interests are also provided (insets).

**Figure 4:**
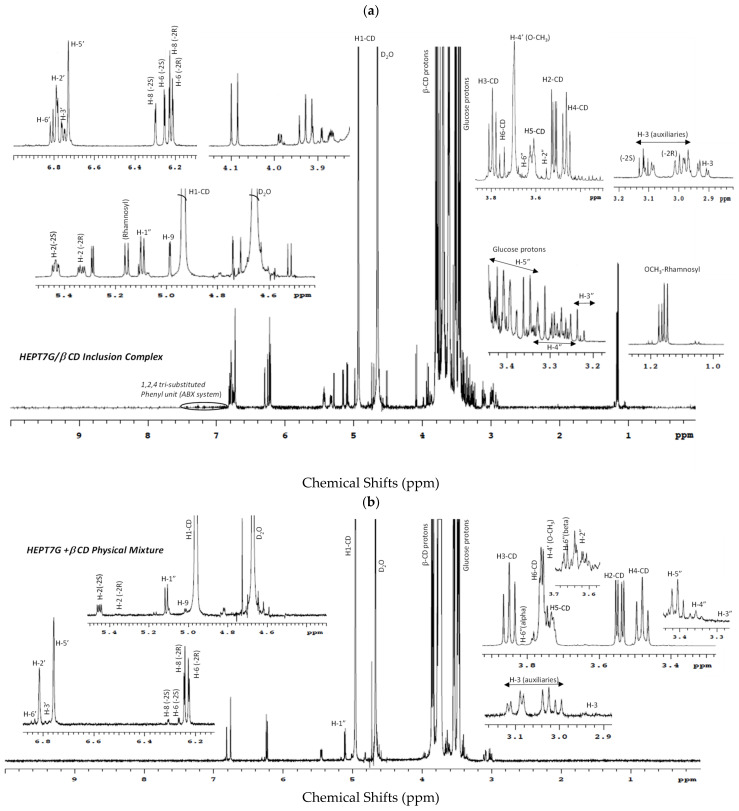
^1^H-NMR spectra of (**a**) the HEPT7G/βCD inclusion complex and (**b**) the HEPT7G+βCD physical mixture are compared. The chemical shift ranges of well-resolved resonance peaks of associated protons of main interests are also described (insets).

The complete ^1^H-NMR spectra of β-CD, pure HEPT7G (standard), and hesperidin (raw material) are displayed in Figure 3, while the comparative ^1^H-NMR spectra of HEPT7G+βCD physical mixture and HEPT7G/βCD inclusion complex are depicted in Figure 4. The ^1^H-NMR peak assignments of all samples, and the chemical shifts of β-CD protons in the absence or presence of HEPT7G moiety were examined and are listed in Table 2. The ^1^H-NMR spectra of pure HEPT7G (standard) and hesperidin were not fully resolved, possibly due to their high crystallinity and poor solubility (see Figure 3). As can be seen from Table 2, the physical mixture of β-CD with HEPT7G (standard) had a negligible effect on the δ values of the β-CD protons (≤0.008 ppm) and also revealed the well-resolved NMR spectrum (see Figure 4). Therefore, to explore the inclusion properties in the HEPT7G/βCD inclusion complex, the chemical shift of β-CD protons and HEPT7G moiety protons were compared with the HEPT7G+βCD physical mixture for the confirmation of the complexation. In the chemical structure of the β-CD molecule, the H3 and H5 protons are located inside the truncated conical cavity, wherein the H3 protons are placed near the wider rim while H5 protons are placed near the narrow rim of the β-CD. Other H1, H2, and H4 protons are located at the exterior of the torus of the β-CD molecule. The H6 protons are located on the border periphery of the cavity rim at the narrow end of the β-CD as illustrated in Figure 1. As shown in Table 2, the presence of the HEPT7G moiety caused an up-field shift in the chemical shifts for almost all protons of β-CD due to the diamagnetic anisotropy of the HEPT7G moiety that inevitably affects the electronic density of the aromatic ring components. Especially noteworthy is the large up-field shift which occurs in the signals for H3 (−0.057 ppm; triplet, t) and H5 (−0.126 ppm; doublet–triplet, dt) protons of β-CD after inclusion complexation, which was probably due to the relatively strong interactions of the hydroxyl groups in the narrow and wide rims of β-CD with the HEPT7G moiety. Both H3 and H5 protons are located in the interior of the β-CD cavity and experience large changes in shielding due to the strong ring-current effect generated by the circulating *π*-electrons (magnetic anisotropy) of the aromatic HEPT7G moiety; therefore, this phenomenon indicates that the less polar portion of the HEPT7G moiety should have penetrated the β-CD cavity from the wide rim side. In contrast, the magnitude of the chemical shift of H1 (−0.028 ppm; doublet, d), H2 (−0.017 ppm; doublet–doublet, dd), H4 (−0.007 ppm; triplet, t), and H6 (−0.025 ppm; doublet–doublet, dd) protons of β-CD exhibited relatively weak changes of a marginally smaller extent by the inclusion process which could have been caused by the weak van der Waals forces, such as hydrogen bonding.

The regions of the ^1^H-NMR spectra of hesperidin, HEPT7G, and its inclusion complexes with β-CD are complicated to analyze because it is very challenging to interpret glycosidic chain protons. The chemical shifts of the hesperidin, HEPT7G (standard), and HEPT7G in its β-CD inclusion complex are summarized in Table 3. The pyran protons (H-2 and H-3) associated with the HEPT7G moiety along with aromatic protons (H-8, H-6, H-2′, H-3′, and H-6′) were possible to identify by their chemical shift and coupling constant. The H-1″ proton of pyranose of the glucose ring was de-shielded at 5.10 ppm, due to the effect of two exceedingly electronegative nuclei oxygen, and the splitting pattern was a doublet due to the presence of the neighboring H-2″ proton at 3.57 ppm. The chemicals of the other glucose ring protons (H-3″, H-4″, H-5″ and H-6″) were assigned to the HEPT7G/βCD spectrum of the inclusion complex, whereas the rutinose proton population in hesperidin was merged at 3.2 to 4.2 ppm in the proton NMR spectrum, as illustrated in Figure 3 and Figure 4 (standard numbering system was used for flavonoids).

The pyranose protons H-3 appeared at 2.92 ppm in the HEPT7G/βCD inclusion complex spectrum, which corresponds to a doublet–doublet due to the presence of two stereospecific protons H-2 and auxiliaries H-3 proton. However, no peaks of H-3 auxiliaries’ protons were observed in the hesperidin (raw material) NMR spectrum, indicating that H-3 and H-2 are almost in near-planner configuration. This means that the B-ring of hesperidin is an equatorial position that confirms the –2S isomeric configuration form of hesperidin [72,73,74]. It is also reported that hesperidin extracted from the *Citrus reticulata* generally exhibits a –2S isomer at room temperature due to its acidic media [72]. In contrast, the results suggest a change in the environment of the aromatic protons (H-8 and H-6) of the ring-A of HEPT7G moiety as a consequence of its interaction with the β-CD molecule. A close observation of the ^1^H-NMR spectrum of the HEPT7G/βCD inclusion complex revealed the presence of another flavanone-like molecule. The presence of side signals close to the signals of H-8 and H-6 protons at 6.23 ppm and 6.21 ppm, respectively (corresponding to doublet), together with the splitting pattern of H-2 proton at 5.28 ppm (corresponding to a doublet of doublet), indicated an isomer of the HEPT7G moiety (i.e., a diastereomeric form of HEPT7G) present in the proprietary HEPT7G/βCD inclusion complex. From the two sets of proton signals detected in the HEPT7G/βCD inclusion complex spectrum, both epimeric flavanone glucosides were characterized for their typical flavonoid structure with resonance at δ 6.30 (d, J_H_ = 2.4 Hz, H-8), δ 6.25 (d, J_H_ = 2.1 Hz, H-6), δ 5.43 (dd, J_H_ = 7.9 Hz, H-2), δ 3.11 (m, J_H_ = 17.1 Hz, H-3_aux_) for –2S isomeric form, and at δ 6.23 (d, J_H_ = 2.3 Hz, H-8), δ 6.21 (d, J_H_ = 2.0 Hz, H-6), δ 5.28 (dd, J_H_ = 10.2, H-2), δ 2.99 (m, J_H_ = 19.2, H-3_aux_) for –2R isomeric configuration, respectively. In the case of the HEPT7G+βCD physical mixture, the ^1^H-NMR spectrum revealed that –2S isomers of HEPT7G tend to convert to the –2R isomeric form thorough the thermal–mechanical transformations that occur during the mixing of pure HEPT7G (standard) and β-CD. As compared with the HEPT7G/βCD inclusion complex, a much higher level of the –2R isoform of the HEPT7G moiety (nearly 90.3%) was detected without significant interactions with inner and exterior β-CD protons (for more information, please refer to Table 3). The ratio of the two stereoisoforms of the HEPT7G moiety estimated from average counts of relative peak intensities of corresponding H-8 and H-6 protons in the ^1^H-NMR spectrum of the HEPT7G/βCD inclusion complex revealed an approximate –2S/–2R epimeric ratio of approximately 1/1.43 (i.e., –2S: 41.1% and –2R: 58.9%) in the inclusion complex. The results presented in Table 3 also suggest a change (shielding) in the surroundings of the protons H-2′ (6.79 ppm, doublet), H-3′ (6.76 ppm, doublet–doublet), OCH_3_-4′ (3.70, singlet), OH-5′ (6.73 ppm), and H-6′ (6.81 ppm) of the aromatic ring-B of the HEPT7G moiety as a consequence of its interaction (possibly an α-orientation) with β-CD. The complete assignment of the ^1^H-NMR spectral signals of the two isomeric structures is provided in Table 3.

**Figure 5:**
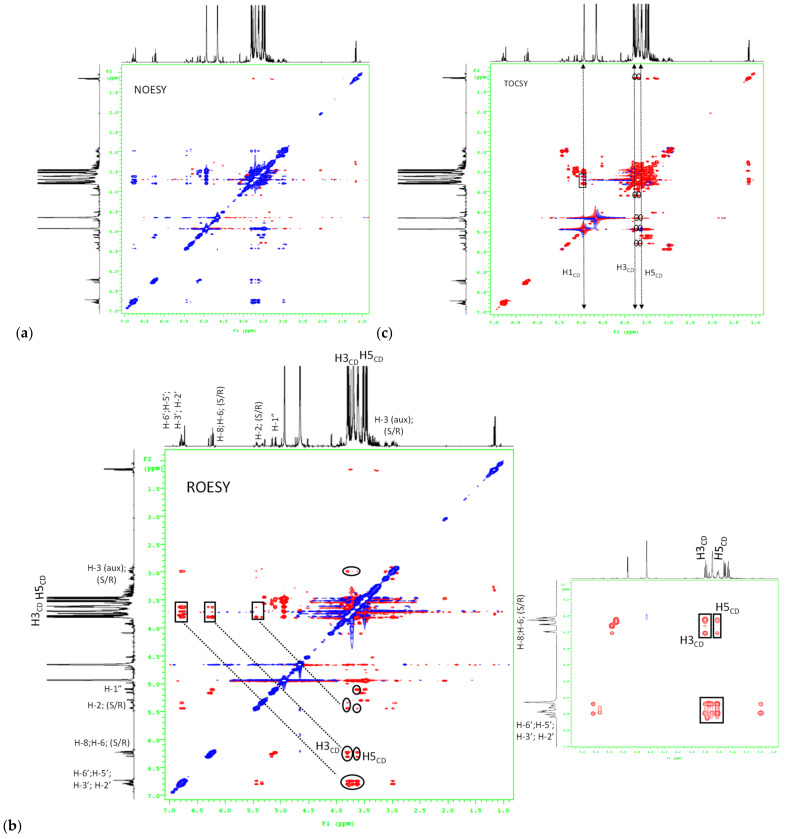
The displayed profiles of two-dimensional (**a**) 2D−NOESY, (**b**) 2D−ROESY, and (**c**) 2D−TOCSY proton nuclear magnetic resonance spectra of the HEPT7G/βCD inclusion complex confirm the correlation between sets of protons of the HEPT7G moiety and the β–CD molecule associated with the complexation phenomenon. The correlated crossed peaks between two protons of the β–CD molecule and the HEPT7G moiety are highlighted in the box and circled in the 2D−NMR spectra.

To gain further conformational information, we employed two-dimensional (2D) NMR spectroscopy, which provided additional evidence about the spatial proximity between β-CD and the HEPT7G moiety through the observations of intermolecular dipolar cross-correlations [59,75,76,77]. Nuclear Overhauser effect (NOE) spectroscopy measurements (NOESY) provide useful information about the supramolecular topology and are important tools to affirm the HEPT7G moiety inclusion in the β-CD cavity. Moreover, rotating frame Overhauser effect spectroscopy (ROESY) has been used to establish the stereochemistry of various flavonoids [71]. Any two protons that are located within 4 Å in space can produce an NOE cross-correlation in NOESY or ROESY [78,79]. The ROESY and NOESY experimental spectra are illustrated in Figure 5, confirming the inclusion mode of the HEPT7G moiety with β-CD. The NOEs detected in the 2D-NOESY experiment cross-peak correlation between the aromatic protons H-8, H-6, H-2′, H-3′, H-5′, H-6′ (i.e., A and B ring protons), pyran protons H-2, H-3 _auxiliary_ (i.e., C ring protons), including glucosidic protons of the HEPT7G moiety and protons H-3 and H-5 of the β-CD inner cavity, confirm the formation of the HEPT7G/βCD inclusion complex. A similar inference was derived from the 2D-ROESY cross peak correlation spectrum of the HEPT7G/βCD inclusion complex, apparently revealing that aromatic protons of the HEPT7G moiety are strongly correlated with both H-3 and H-5 protons located inside of the β-CD cavity; we deduce that the HEPT7G moiety was completely included in the β-CD cavity from the wider rim side. The glucoside protons also showed a moderate correlation with H-3 and H-5 protons in the β-CD inner cavity; therefore, their inclusion was confirmed in the inner cavity of the β-CD. Based on these observations, the hypothesis of 1:1 complex stoichiometry of the HEPT7G/βCD inclusion complex could be supported because all specific sites of the HEPT7G moiety presented dipolar correlations with the β-CD molecule (as observed in the 2D-ROESY and 2D-NOESY contour maps); thus, we postulated the possible inclusion mode of the HEPT7G moiety with the β-CD molecule, as illustrated in Figure 1. We also performed two-dimensional total correlation spectroscopic (2D-TOCSY) cross-peak measurements of the HEPT7G/βCD inclusion complex (see Figure 5c), where correlations for protons of the same glucose unit can be easily distinguishable from different glucose units. Although 2D-TOCSY cross-peak intermolecular interactions are distinct in the phase-sensitive spectrum of the HEPT7G/βCD inclusion complex, they have contrasting signs of 2D-ROESY cross-peaks. Furthermore, 2D-TOCSY cross-peak measurements create a correlation between all protons within a given spin system that stabilize the complexation with relatively weak molecular interactions (van der Waals interaction, hydrogen bonding, or electrostatic forces), and thus clearly support the inference of HEPT7G/βCD inclusion complex formation.

**Figure 6:**
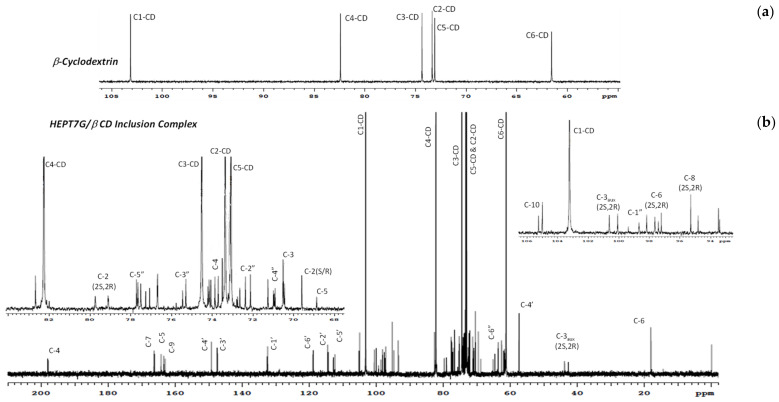
^13^C-NMR spectra of (**a**) β–CD and (**b**) the HEPT7G/βCD inclusion complex. The chemical shift ranges of well-resolved resonance peaks of associated carbons of main interests are also described (insets).

The representative ^13^C-NMR spectra of the HEPT7G/βCD inclusion complex and the free β-CD are depicted in Figure 6, clearly revealing the intense resonance of all six carbon atoms associated with the β-CD molecule. The typical assignment of the resonance of carbon atoms associated with the HEPT7G moiety in the HEPT7G/βCD inclusion complex spectrum is listed in Table 3. In addition, the related chemical shifts corresponding to β-CD in the presence or absence of the HEPT7G moiety are presented in Table 4. The changes in the ^13^C-NMR chemical shifts of β-CD carbons caused by the proximity of the host and guest in the complexation process indicate that the HEPT7G moiety entered the β-CD cavity and evidenced the formation of the HEPT7G/βCD inclusion complex. The introduction of the HEPT7G moiety in the β-CD cavity results in a mild high-frequency shift of C1, C2, and C3, whereas chemical shifts of C4, C5, and C6 move in the opposite direction (i.e., toward a lower frequency position). ^13^C NMR shifts extended over a much larger scale than proton shifts, particularly for C1, C3, C4, and C6, which evidenced the existence of an interaction between the HEPT7G moiety and the interior of the host β-CD cavity with complete inclusion; hence, the successful complexation. The results reveal the systematical conformation of HEPT7G moiety inclusion inside the proprietary HEPT7G/βCD inclusion complex, which is in accordance with ^1^H NMR results.

### 3.3. Thermal Analysis of the Complexation

**Figure 7:**
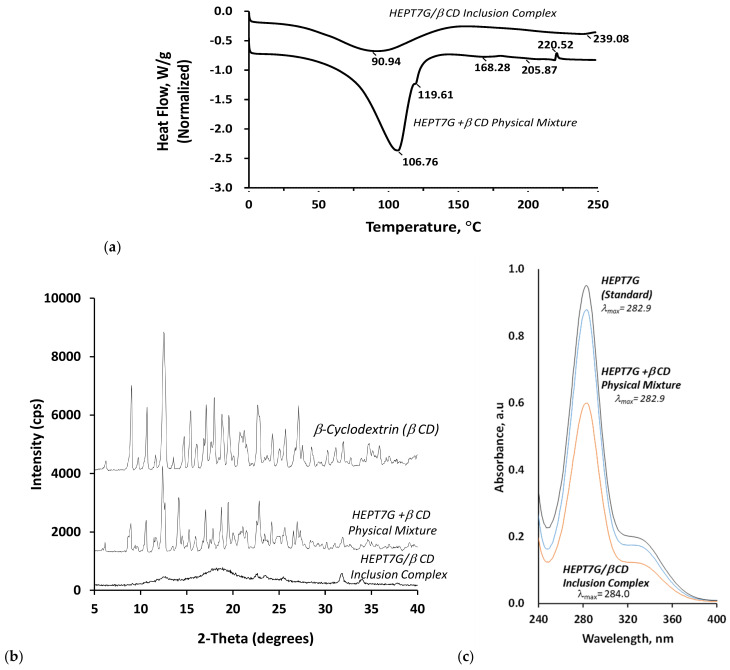
(**a**) Differential scanning calorimetry (DSC) thermogram comparing the HEPT7G+βCD physical mixture and HEPT7G/βCD inclusion complex. (**b**) X-ray diffraction profile comparing the crystal phases of β–CD, the HEPT7G+βCD physical mixture, and the HEPT7G/βCD inclusion complex. (**c**) UV–Vis absorption spectra in aqueous solution comparing the pure HEPT7G (standard), HEPT7G+βCD physical mixture, and HEPT7G/βCD inclusion complex.

The differential scanning calorimetry (DSC) thermographs collected for the HEPT7G/βCD inclusion complex and HEPT7G+βCD physical mixture are presented in Figure 7a; their thermal stability was assessed by the differences that occurred via phase transformations during the heating profile. The results provide useful insight into the solid-state interactions between the β-CD and HEPTG components. In the case of the HEPT7G+βCD physical mixture, endothermic dehydration peaks were observed at 106.7 and 119.6 °C, corresponding to the elimination of free water and crystalline water from the β-CD cavity, respectively. In the HEPT7G/βCD inclusion complex, however, the endothermic dehydration peak with a marked broadening of less intensity was significantly shifted toward lower-temperature domains centered at 90.9 °C, as a result of the molecular inclusion of the HEPT7G moiety in the β-CD inner cavity. Additionally, a significant decrease in the dehydration peak enthalpy value of the HEPT7G/βCD inclusion complex (168.54 J/g; onset: 39.2 °C) was observed compared with the HEPT7G+βCD physical mixture (340.0 J/g; onset: 39.0 °C).

HEPT7G (standard) and β-CD exhibited individual endothermic peaks at 206.1 and 268.3 °C, respectively, corresponding to their melting heat value tendency (spectra not shown). The broad endothermic peaks observed at 168.3 and 205.9 °C in the DSC thermogram of the HEPT7G+βCD physical mixture were due to the decomposition of respective HEPT7G and β-CD components. In the case of the HEPT7G/βCD inclusion complex, all the prominent peaks belonging to HEPT7G and β-CD decomposition completely disappeared, and a characteristic broad endothermic peak emerged at 239.1 °C (see Figure 7a). Such differences not only demonstrate the occurrence of the complexation phenomenon, but also suggest the stability of the HEPT7G/βCD inclusion complex. The observed broadening and shifting toward the higher temperature of the endothermic peak of the HEPT7G/βCD inclusion complex could be attributed to the phenomenon of masking the HEPT7G moiety melting endotherm and/or the fusion between HEPT7G moiety melting and β-CD thermal transition due to the overlapping vicinity of both effects. Additionally, the substitution of water molecules located in the vicinity of the β-CD inner cavity by the HEPT7G moiety significantly affects the van der Waals interactions and/or hydrogen bonding, resulting in stable complex formation from a thermal viewpoint. The results indicate that HEPT7G crystallization could be prevented by the molecular-level interactions and the altered crystal lattice structure of the solid-state system, confirming that the HEPT7G moiety is embedded in the β-CD cavity to form a stable amorphous (bulk type structure) HEPT7G/βCD inclusion complex.

### 3.4. X-Ray Crystallography Studies of Complexation

To investigate the molecular crystalline/amorphous morphology of hesperetin-7-*O*-glucoside after inclusion complex formation, the powder X-ray diffractometric (PXRD) analysis patterns of β-CD, the HEPT7G/βCD inclusion complex, and the HEPT7G+βCD physical mixture were determined and are displayed in Figure 7b. As can be observed from the PXRD patterns, physically mixed HEPT7G with β-CD (i.e., HEPT7G+βCD sample) is approximately the superposition of the patterns of the raw materials, indicating no new solid-state domain formation, and the diffractogram is consistent with the crystalline nature of HEPT7G and β-CD. The β-CD diffraction pattern exhibited well-distinguished sharp characteristic peaks at the 2θ values of 6.23°, 9.01°, 10.68°, 12.50°, 14.67°, 15.29°, 15.42°, 17.10°, 17.65°, 18.93°, 19.59°, 21.21°, 22.70°, 24.27°, 25.66°, 27.10°, 28.54°, 30.99, 31.95°, 34.74°, 35.86°, and 36.92°, indicating the crystalline domains of β-CD morphology. Similarly, the HEPT7G+βCD physical mixture displayed characteristic crystalline peaks at the 2θ values of 6.14°, 8.92°, 10.58°, 12.40°, 14.14°, 15.28°, 17.03°, 17.65°, 18.69°, 19.51°, 21.05°, 22.84°, 24.19°, 24.77°, 25.43°, 26.96°, 28.63°, 31.89°, 34.61°, and 36.91°; the sum of the HEPT7G and β-CD and did not show particular characteristic differences. Assessment of the diffraction peaks with relative intensities nearly 30% and above revealed that the HEPT7G +βCD physical mixture displayed a slight shift to either lower or occasionally higher Bragg diffraction angles (2θ) along with moderately decreased intensities without any particular damage to the basic skeleton of the β-CD cavity or new phase formation.

A broad halo diffraction pattern of the HEPT7G/βCD inclusion complex confirmed its amorphous nature (similar to bulk), wherein diffraction peaks are obviously different. All crystalline peaks almost disappeared, and it is no longer possible to distinguish the characteristic peaks of the superimposed crystal morphological feature of hesperetin-7-*O*-glucoside (see Figure 7b). The results indicate that HEPT7G moieties are completely included in the β-CD and reoriented in the β-CD inner cavity, thereby increasing the dissolution rate of the HEPT7G moiety during the HEPT7G/βCD inclusion complex formulation, as the differences in the interplaner spacing, relative diffraction peak intensities and diffraction angles observed at the 2θ values of 12.67°, 22.63°, 23.58°, 25.48°, 31.80°, 33.99°, and 37.84° confirm that the HEPT7G/βCD inclusion complex has amorphous morphological structural phases. The results can be attributed to intermolecular interactions between system components, thus confirming the formation of the HEPT7G/βCD inclusion complex, as indicated by DSC, FTIR, FT–Raman, and NMR spectroscopic studies. Table 5 presents a detailed list of the corresponding PXRD diffraction peaks, their Bragg angles (2θ; degree), relative intensities (I/I_0_; %), and interplanar distances (d; nm).

### 3.5. UV–Vis Spectroscopy Analysis of Complexation

Figure 7c illustrates the UV–Vis absorbance spectra of the reference standard HEPT7G, the physical mixture of HEPT7G with β-CD, and the HEPT7G/βCD inclusion complex in an aqueous solution. The pH of the solution did not change appreciably (nearly 6.0) during any of the experimental procedures. The concentration of HEPT7G was identical in both the physical mixture of HEPT7G with β-CD and the HEPT7G/βCD inclusion complex. The characteristic absorption spectrum of reference standard HEPT7G in the UV–Vis range of 220–400 nm exhibited an intense Gaussian peak at 282.9 nm (*λ*_max1_) and a broad shoulder around 332.3 nm (*λ*_max2_), corresponding to the ‘K’ absorbance (i.e., band II) and ‘B’ absorbance (i.e., band I) bands of the aromatic chromophore and conjugated double bonds in flavonoids associated with two different excited states of molecules ascribed to the A–C ring benzoyl system and B ring of HEPT7G, respectively. These peaks arise due to the n→π* electronic rearrangement of molecules and the n→π* electronic transition of a non-bonding (n) molecular orbital to an antibonding (π*) molecular orbital [80,81,82]. The effect of β-CD on the UV–Vis spectrum of HEPT7G, as displayed in Figure 7c, showed the changes in the wavelength maximum of the absorption band to a longer wavelength (from 282.9 to 284.0 nm) in the UV–Vis absorbance spectra of the HEPT7G/βCD inclusion complex due to a weak bathochromic effect in the presence of β-CD. Additionally, the UV–Vis spectra revealed that the HEPT7G/βCD inclusion complex has the same chromophoric group as reference standard HEPT7G, accompanying a weak bathochromic shift (*λ*_max1_ = 284.0 nm), and the absorption intensity was decreased upon HEPT7G complexation with β-CD, attributable to the possible effect of steric hindrance factors. A small shift was observed in the ‘B’ absorbance band (*λ*_max2_ = 339.2 nm), possibly due to the complexation between HEPT7G and β-CD. These modifications can indicate the successful formation of an inclusion complex. On the other hand, the UV–Vis spectra of the physical mixture of HEPT7G with β-CD were similar to the reference standard HEPT7G with a somewhat lower intensity of the ‘K’ absorption band (*λ*_max1_ = 282.9 nm). This can be attributed to a weak hypochromic effect without any shift of wavelength maximum occurring in the presence of β-CD (see Figure 7c). Additionally, it may be noted that not much change was observed in the ‘B’ absorbance band (*λ*_max2_ = 332.5 nm) during the physical mixing. The similarities of UV–Vis spectra further revealed that the HEPT7G moiety formed by the controlled enzymatic hydrolysis of hesperidin in the presence of β-CD, resulting in the formation of an HEPT7G/βCD inclusion complex without undergoing any thermolysis, photolysis, or degradation byproduct formation.

### 3.6. Scanning Electron Microscopic Analysis

**Figure 8:**
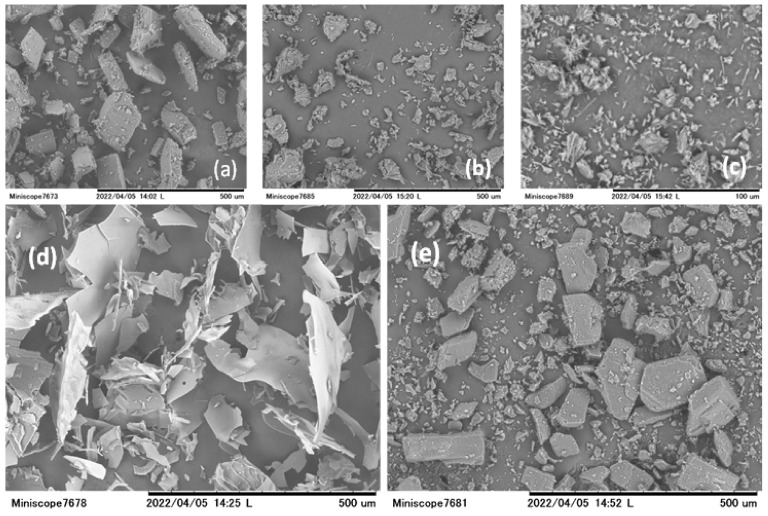
Scanning electron micrographs comparing the crystal properties of (**a**) β–CD, (**b**) pure HEPT7G (standard), (**c**) hesperidin (raw material), (**d**) the HEPT7G/βCD inclusion complex, and (**e**) the HEPT7G+βCD physical mixture.

The bulk surface morphologies of the particles of the HEPT7G/βCD inclusion complex were qualitatively observed to determine the structural aspects of the complexation phenomenon. Figure 8 illustrates the topography of scanning electron micrographs of HEPT7G (standard), β-CD, hesperidin (raw material), the HEPT7G+βCD physical mixture, and the HEPT7G/βCD inclusion complex. It is clearly observable in the SEM monographs that β-CD has a bulk crystal form with flat columnar chunk-type morphology. Typical crystals of pure HEPT7G (standard) are found in many different irregular shapes and sizes. However, the raw material hesperidin exhibited a quite different morphologic form, appearing as a tiny needle-type crystal. The SEM image of the physical mixture of HEPT7G with β-CD (HEPT7G+βCD) revealed some similarities with a crystal of both parent pure HEPT7G (standard) and β-CD molecules, and showed both crystalline components without notable differences. However, a drastic change appeared in the shape and morphological features of the HEPT7G/βCD inclusion complex, which was quite different from the shape and size of hesperidin and β-CD. The SEM image of HEPT7G/βCD inclusion complex exhibited thin sheet-like amorphous flakes with smooth surfaces of varied sizes, including flakes with cracks on the surface and pieces with limited aggregation. The original morphology of both raw materials completely disappeared (see Figure 8), and the homogenously distributed new solid-phase morphology confirmed the occurrence of the inclusion complexation process, indicative of the formation of the amorphous HEPT7G/βCD inclusion complex.

### 3.7. LC– MS/MS Study Results

**Figure 9:**
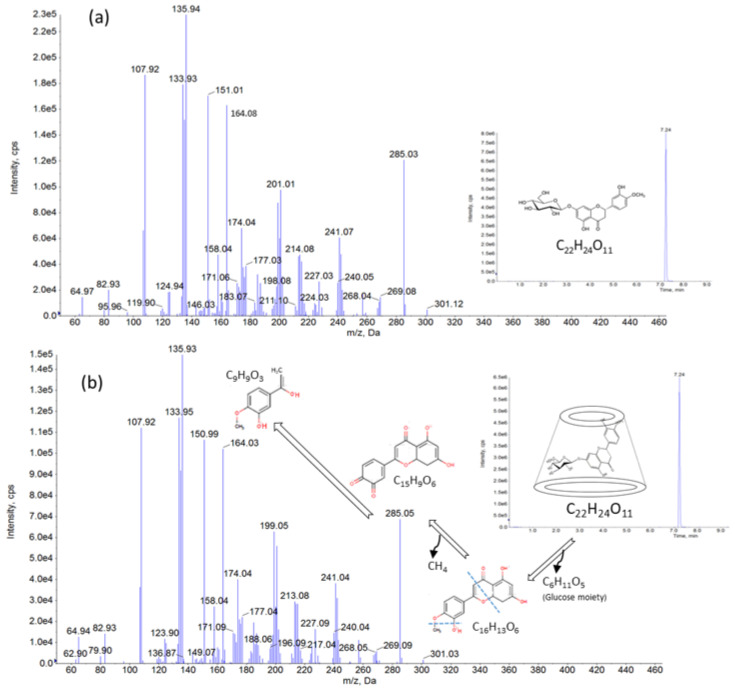
LC–MS/MS extracted ion chromatogram of (**a**) pure HEPT7G (standard) and (**b**) the HEPT7G/βCD inclusion complex for the *m*/*z* 301 ions obtained in negative ion mode. LC–MS chromatogram confirmed an identical retention time of elution (7.24 min) for the free HEPT7G moiety and the HEPT7G moiety embedded with the HEPT7G/βCD inclusion complex (insets).

The molecular transition *m*/*z* 301.0 to *m*/*z* 163.8 and to *m*/*z* 150.8 were used to determine the reference standard of HEPT7G and the HEPT7G/βCD inclusion complex (SunActive^®^ HCD) using negative ion electrospray ionization (ESI) by LC–MS/MS. The method was validated according to U.S. Food and Drug Administration bioanalytical method validation guidelines for industry [83] to confirm its sensitivity, selectiveness, accuracy, and repeatability for the quantification of samples analyzed. A single peak appeared at 7.24 min in the chromatographic profile of the HEPT7G/βCD inclusion complex, which was the same retention time as the peak obtained from the reference standard of HEPT7G. In addition, the total ion current (TIC) profile of the standalone peak at the retention time (7.24 min) obtained from the HEPT7G/βCD inclusion complex was quite similar to that of the peak at the same retention time obtained from an HEPT7G reference standard (see Figure 9). The observed peak intensities of the HEPT7G/βCD inclusion complex were somewhat lower compared with standard HEPT7G reference due to complexation with β-CD. In the LC–MS/MS spectra of the HEPT7G/βCD inclusion complex, the peak eluted at 7.24 min, giving rise to the deprotonated molecule at *m*/*z* 301.03 [M–H], indicating the presence of aglycones of hesperetin, which was proposed as being formed through the loss of glucose from the ion at *m*/*z* 464.4 (i.e., monoisotopic mass of hesperetin-7-*O*-glucoside), thus confirming the presence of the HEPT7G moiety in the HEPT7G/βCD inclusion complex. In the precursor/product ion pair investigation, the fragmentation of the precursor at *m*/*z* 301.03 exhibited major intense product ions at *m*/*z* 285.05 and *m*/*z* 164.03 including *m*/*z* peaks at 241.04, 213.08, 199.05, 174.04, 150.99, 135.93, and 107.92. The LC–MS/MS spectra of the HEPT7G reference standard also showed a similar deprotonated molecule at *m*/*z* 301.12 [M–H], whose fragmentation led to the product ions at *m*/*z* 285.03, and *m*/*z* 164.03, including *m*/*z* peaks at 241.07, 214.08, 201.01, 174.04, 151.01, 135.94, and 107.92 (see Figure 9). Thus, the LC–MS/MS results further support the successful formulation of the HEPT7G/βCD inclusion complex.

### 3.8. The Zeta Potential Studies

The zeta potential, which is an indicator of surface charge, revealed negative zeta potential values of β-CD (−23.7 mV) due to free hydroxyl surface groups. The negative charge at the surface indicated that molecular aligning on the amphiphilic β-CD arranged with the unsubstituted hydroxyl groups was directed towards the aqueous surrounding, rendering a potential surface hydrophobicity [84]. The zeta potential values of pure HEPT7G and HEPT7G/βCD inclusion complex particles were −22.1 and −26.9 mV, respectively. The charge that HEPT7G moiety distributed increased the mass of the electron cloud, which led to the increased stability of the HEPT7G/βCD inclusion complex particles. The results revealed that the zeta potential was lowered when the HEPT7G moiety was included in the β-CD cavity. This can be attributed to the blocking of the –OH group by the HEPT7G moiety inclusion through an electrostatic attraction between the acidic phenolic –OH groups of the HEPT7G moiety and the charge-influenced distribution of β-CD particles [85,86]. Therefore, an enhancement in the surface charge could be attributed to the formation of hydrogen bonding between the β-CD and charged HEPT7G moiety head groups. Thus, zeta potential may define the higher degree of stability and solubility HEPT7G/βCD inclusion complex and govern the synergetic enwrapping of the homogeneously distributed charged HEPT7G moiety with negatively charged β-CD.

### 3.9. Solubility Measurement, Job’s Plot, and Phase–Solubility Studies

**Figure 10:**
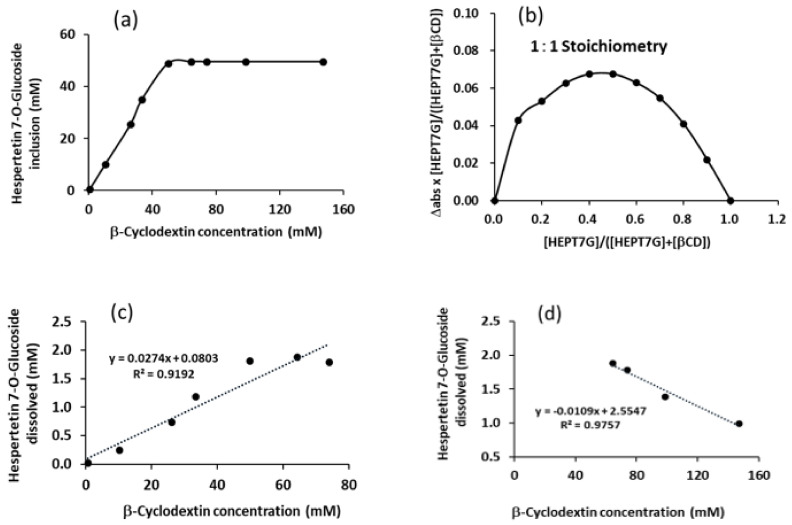
(**a**) Profile of the HEPT7G inclusion rate into the β–CD cavity for HEPT7G/βCD inclusion complex formulation, and phase–solubility curves of the HEPT7G/βCD inclusion complex of varied HEPT7G and β–CD ratios, (**b**) equimolar ratio and below, (**c**) equimolar ratio and above; in aqueous solution at ambient conditions (at 25 °C), and (**d**) Job’s plot of the HEPT7G/βCD system at ambient conditions (at 25 °C; *λ*_max_ = 282–284 nm). Δ _abs_ = difference in absorbance of the HEPT7G moiety without and with β–CD; R _concentration_ = [HEPT7G]/{[HEPT7G] + [β–CD]}.

To evaluate the solubility of the HEPT7G/βCD inclusion complex system, dissolution studies were performed. An increase in HEPT7G aqueous solubility as a function of the β-CD concentration was observed in the range of 0–70 mM upon the complexation of HEPT7G with β-CD. The estimated aqueous solubility of pure HEPT7G (standard) was found to be 0.007%, classifying it as practically insoluble. The solubility of HEPT7G was remarkably higher in water (3.8%) upon inclusion in the cavity of β-CD after complexation, which was approximately 500-fold greater than the HEPT7G (standard). An enhancement in the water solubility could be attributed to the effect of blocking lipophilic sites as a result of HEPT7G inclusion in β-CD, and an increase in the number of hydroxyl groups associated with a β-CD solubility effect. The hydrolysis of the rhamnosyl moiety from hesperidin resulted in the formation of HEPT7G, which improved its water solubility. Furthermore, the rate of HEPT7G moiety inclusion into β-CD, depicted in Figure 10a, reveals the progressive formation of the HEPT7G/βCD inclusion complex after cleaving the rhamnosyl unit from hesperidin. The β-CD cavity is suitable for the inclusion of common chemical molecules with molar masses in the range of 200 to 800 Da (i.e., g.mol^−1^) [58,59]. Although a 1:1 stoichiometry was determined for the HEPT7G/βCD inclusion complex, an excess of β-CD was employed to increase the solubility as well as the yield of complexation. Similar strategies have been reported elsewhere in the literature [70]. The 1:1 stoichiometry of the HEPT7G/βCD inclusion complex was further confirmed with the Job’s plot method (also defined as the continuous variation method) using UV–Vis spectroscopy to measure the absorbance values at *λ*_max_ (285 nm) of the respective solutions at ambient temperature. A set of solutions for HEPT7G moiety and β-CD was prepared with varying amounts of mole fraction of the HEPT7G in the range (R) 0 to 1. The Job’s plot was obtained by plotting Δ _abs_ × R _concentration_ against R _concentration_, wherein Δ _abs_ is the difference in absorbance of the HEPT7G moiety with and without β-CD, and R _concentration_ = [HEPT7G]/{[HEPT7G] + [β-CD]}. The value of R _concentration_ at the maximum deviation for the observed Job’s plot was found at 0.5, thus revealing the 1:1 stoichiometry of the HEPT7G/βCD inclusion complex (see Figure 10b). Furthermore, a linear growth in the aqueous solubility of HEPT7G was noticed with increasing concentrations of β-CD until the equimolar ratio was attained. The linear positive isotherm presented in Figure 10a has a slope of less than 1 (i.e., m = 0.565) and can be classified as an A_L_ type, suggesting the formation of a 1:1 inclusion complex between the HEPT7G moiety and β-CD, according to Higuchi and Connors’s model [87,88]. Notably, HEPT7G solubility was increased more than 500-fold upon complexation with β-CD. The solubility limit of the complexation was reached with a further increase in the concentration range of the β-CD. The rate of HEPT7G solubilization was somewhat slow at the beginning because reaction-terminated liquid mixtures were in a suspended state; however, at nearly 100% HEPT7G inclusion, the reaction liquid mixture was homogeneously dissolved at the time of termination of the reaction with an access range of the β-CD concentration.

The apparent stability constant (K_1:1_; binding or association constant) estimated from the linear region of the solubility plot diagram presented in Figure 10c (R^2^ = 0.9755; slope m = 0.0272) using the Higuchi and Connors method for HEPT7G/βCD inclusion complex was 26572 M^−1^ [87,89,90], demonstrating that the process is favorable to obtain inclusion complex. The observed very high apparent stability constant corroborates the moderate complexation efficiency (CE) of the HEPT7G/βCD inclusion complex (CE = 281.7), indicating that a relative excess amount of β-CD was necessary to solubilize a reasonable amount of HEPT7G in aqueous media to achieve solubilization limits. Complexation efficiency is the concentration ratio between β-CD in a complex and free β-CD, is independent of both S_0_ and the intercept, and is estimated from the slope of the phase–solubility diagram. The intrinsic solubility (S_0_) of HEPT7G determined under identical reaction conditions was about 0.0106 mM, whereas the stability constant after the solubility limit of HEPT7G was reached was estimated from the non-linear region of the phase–solubility plot (see Figure 10d), indicating a negative slope (*m* = −0.0109; *R^2^* = 0.9757), and the solubility constant was found to be 10396 M^−1^. This result revealed that at higher β-CD concentrations, the β-CD molecules might undergo self-assembling to form aggregates, wherein the increased aggregation rate and the size of the aggregates resulted in a decrease in the HEPT7G solubility of HEPT7G/βCD inclusion complex. Such a decrease in the solubilizing effect of β-CD in HEPT7G/βCD inclusion complex at higher β-CD concentrations could be further supported by the complexation efficiency (CE = 110.2) and considered reliable when the influences of excipients on the solubilization are investigated. Based on the phase solubility studies, a very high stability constant obtained for the HEPT7G/βCD inclusion complex again confirmed the favorable conditions for the formation of a highly soluble complex with 1:1 stoichiometry.

Hesperetin-7-*O*-glucoside has a molecular formula C_22_H_24_O_11_ (molecular weight: 464.4 g/mol; CAS: 2500-68-7); its chemical structure is displayed in Figure 1. In the formulation of the proprietary HEPT7G/βCD inclusion complex, along with the steric factors, the interactions between several components of the system (cyclodextrin, inclusion substance, and solvent), including size–shape fit, are critical dominant controlling factors in the formation of an inclusion complex having enhanced solubility of compounds that are barely soluble in water for a wide range of therapeutic activities. Favorable energy is required to push the HEPT7G moiety into the β-CD cavity, and generated when the reaction temperature gradually regulates the complexation between the HEPT7G moiety and β-CD. In this viewpoint, the thermodynamic parameters of complexation reactions also play an important role, along with stability and solubility constants, wherein the binding process is dependent on the net entropy gain due to the substantial arrangement and expulsion of a high-energy water molecule from the β-CD cavity arising from changes in the translational and conformational freedom upon complexation. This possibly gives rise to an adequate distribution of a polar–apolar association. The reasonably higher spatial distribution of water molecules in the β-CD cavity [91] could be associated with the 1:1 complexation interaction of an HEPT7G moiety with β-CD, also revealed from the Job’s plot, suggesting that the process of HEPT7G moiety inclusion is possibly varied from entropy-driven to an enthalpy–entropy-driven process. Therefore, the formation of the proprietary HEPT7G/βCD inclusion complex was a spontaneous and repeatable process, wherein the prevention of crystallization is a crucial factor in the significant enhancement of HEPT7G solubility. Based on the crystallographic analysis, it is believed that the molecular dimensions of the β-CD cavity (inner diameter 0.60–0.65 nm, external diameter 1.52 nm, and height 0.78 nm) make it the ideal host among native cyclodextrins for inclusion complex formation with the HEPT7G moiety. The topological polar surface area of the HEPT7G molecule is about 175 Å^2^, whereas the cavity volume of β-CD is 262 Å^3^ [92] (Szejtli, 1997). Therefore, the HEPT7G molecule can fit comfortably into the β-CD cavity.

In this paper, our approach aimed to characterize the proprietary HEPT7G/βCD inclusion complex and provide evidence that inclusion complexation has an impact on the stability, dissolution, and solubility of sparingly soluble HEPT7G moiety, which can further be translated into better bioavailability. FT–Raman and FTIR studies confirmed the formation of the HEPT7G/βCD inclusion complex. It also seems likely that changes observed in the PXRD patterns (i.e., absence of noticeable crystalline peaks) and lack of the endothermic peak corresponding to dissociation of complex components in the DSC thermogram suggested the successful formation of the HEPT7G/βCD inclusion complex. Interestingly, the physical mixture provided little dissolution improvement, but it was not suitable enough as in the case of the amorphous HEPT7G/βCD inclusion complex. In addition to other techniques that rationalized several physicochemical properties of the HEPT7G/βCD inclusion complex, the NMR spectroscopic method offered the main conclusive evidence and suggested the decisive structural complexity of the HEPT7G/βCD inclusion complex. Based on the ^1^H NMR results with the assistance of two-dimensional (2D) NMR experiments, the detailed ^1^H resonance assignments of the isoform of HEPT7G were elucidated to reveal the isomeric composition (both –2S and –2R isoforms) of the HEPT7G/βCD inclusion complex. Hesperidin extracted from *Citrus reticulata* generally exhibited a –2S isoform at room temperature due to its acidic media [72]. However, it is believed that the involvement of a manifold of enzymes in the biosynthesis of phytochemicals results in the highly stereoselective production of natural flavonoids such as hesperidin and its aglycones. The NMR spectroscopy results presented in this paper clearly distinguished stereoisomers and made it possible to identify the proportional ratio of two epimeric forms of hesperetin-7-O glucoside. Gaffield [93] reported that flavanones of –2S isoform configuration exhibit a positive Cotton effect due to n→π* transition observed at lower-energy wavelengths (325–340 nm), and a negative Cotton effect due to π→π* transition was observed at the maximum absorption wavelength (282–285 nm). Therefore, we assigned an absolute configuration –2R of HEPT7G to the ^1^H-NMR resonances that appeared at lower chemical shifts, and –2S of HEPT7G to the ^1^H NMR resonances observed at higher chemical shifts (see Figure 4). Furthermore, the stereochemistry of the vicinal coupled protons is strongly based on the dihedral angle (ϕ), which is maximum near 0° to 180° or minimum near 90° and 270°. In the hesperetin moiety, the aromatic ring part of benzopyrone and the phenyl ring form the twist orientation (dihedral angle of two rings, ϕ is 53.1°), wherein the pyrone ring forms a slightly flattened sofa conformation [94]. The dihedral angle of these two rings in the hesperidin molecule has been reported at 51.8° [95]. Unfortunately, there are no references for the hesperetin-7-*O*-glucoside molecule. Furthermore, assuming a nearly similar dihedral angle for the HEPT7G moiety for its approximate positioning and orientation, possibly the molecule headed by its phenyl ring (B ring) followed by benzopyrone and chromen structure rings (C and A rings) into the β-CD cavity, and rearranged its positioning due to relatively strong polar–apolar interactions of the hydrophobic inner surface of the β-CD cavity. In addition, due to electron donor characteristics of the hesperetin and its derivatives, their methoxyphenyl as well as planner benzopyrone rings are known to be clustered and form somewhat weak π–π interactions with each other. However, in the HEPT7G/βCD inclusion complex formulation process, the possibility of the clustered association of HEPT7G moiety to form a non-covalent dimer complex could be negligible due to the absence of any strong hydrogen bonding for intermolecular interactions and energy stabilizing between the HEPT7G moieties in the presence of β-CD. The inclusion of the HEPT7G moiety in the β-CD cavity stabilizes its hydroxyl groups (-OH) through spatial hydrogen bonding with hydroxyl groups located within the boundaries of the orbital wall of β-CD, and shielding them in the β-CD cavity. The spectroscopic evidence summarized in this study demonstrates the successful formation of the HEPT7G/βCD inclusion complex with remarkable enhancements in HEPT7G solubility in water. In a single-dose human clinical study, a significantly high bioavailability (>100-fold) of HEPT7G/βCD inclusion complex compared with hesperidin is revealed [96]. It is postulated that HEPT7G is rapidly digested in the small intestine, absorbed efficiently, and released the aglycone that is rapidly glucuronidated and sulfated to achieve a higher plasma concentration of hesperetin metabolites. Furthermore, it is suggested that the biological activity and bioavailability of the HEPT7G/βCD inclusion complex depend not only on its improved solubility, but also on the lipophilicity and hydrophilicity balance that controls its binding capacity, transport activity, interaction with membranes, and their ability to influence the absorption.

## 4. Conclusions

The present study explains that the hesperetin-7-*O*-glucoside moiety obtained via the enzymatic hydrolysis of hesperidin with naringinase enzyme in the presence of β-CD forms a highly soluble inclusion complex of 1:1 stoichiometry with the β-CD molecule in an aqueous medium, which was also confirmed by Job’s plot. The changes in the profiles of analytical spectroscopic features associated with the HEPT7G moiety and β-CD molecule revealed the obvious formation of the HEPT7G/βCD inclusion complex. The apparently high-stability constant (i.e., association constant; 26,572 M^−1^) estimated from the phase–solubility plot using Higuchi and Connors’s method for the HEPT7G/βCD inclusion complex demonstrates that the formulation process is favorable to obtaining an inclusion complex. The moderate complexation efficiency (CE = 281.7) of the HEPT7G/βCD inclusion complex indicates that a relative excess amount of β-CD was necessary to solubilize a reasonable amount of HEPT7G in aqueous media to achieve solubilization limits. The 2D ROESY, NOESY, and TOCSY ^1^H–NMR assays also confirm the successful occurrence of the inclusion phenomenon. The reliable spectroscopic studies with high accuracy indicated the complete encapsulation of the HEPT7G moiety in the β-CD inner hydrophobic cavity. The limitation of this study is the lack of required experimental molecular docking simulation studies to effectively interpret and predict the structural geometry of the HEPT7G/βCD inclusion complex. The presence of both –2S and –2R stereoisomers of hesperetin-7-*O*-glucoside in the –2S/–2R epimeric ratio of 1/1.43 (i.e., –2S: 41.1% and –2R: 58.9%) could also contribute to the enhanced bioavailability of the HEPT7G/βCD inclusion complex for medical health and pharmaceutical applications. In summary, the HEPT7G was successfully produced from hesperidin by an enzymatic reaction and formed a highly soluble inclusion complex with β-CD, which makes the HEPT7G/βCD inclusion complex a more bioavailable and effective functional flavonoid ingredient with potential health benefits in humans.

## Figures and Tables

**Table 1:** FTIR and Raman scattering spectroscopic data of vibrational band assignments (wave number, cm^−1^) of β–CD, pure HEPT7G (standard), HEPT7G+βCD physical mixture, and HEPT7G/βCD inclusion complex.

FTIR Spectrum Wave Number (cm^−1^)				Description of Vibrational Regions	Raman Shift Wave Number (cm^−1^)			
β–*CD*	*HEPT7G Standard*	*HEPT7G/*β*CD Inclusion* *Complex*	*HEPT7G+*β*CD Physical Mixture*	*Band Assignment of Vibrational Modes of* *Functional Groups*	β–*CD*	*HEPT7G* *Standard*	*HEPT7G/*β*CD* *Inclusion Complex*	*HEPT7G+*β*CD Physical* *Mixture*
***Vibrational band frequency region (4000–2500 cm^−1^)***								
	3485			Hydroxyl group (–OH) stretching vibration mode of hydrophobic activity of β-CD bridged system (symmetric and asymmetric phenolic). Primary –OH bonded to ring oxygen intermolecular or to each other and stretching vibration of secondary hydroxyl groups bonded to each other in a different way.	4039	4035	4032	4040
3374	3351	3363	3363					
	3260					3034		
	2972			Aliphatic CH, CH_2_ stretching vibration mode (symmetric and asymmetric bonds); SP3 carbons.		2975		
2926	2929	2930	2928		2911	2928	2930	2938
	2909					2908	2905	2913
						2889, 2840		
***Vibrational band frequency region (1700–1200 cm^−1^)***								
1655				Deformation band of adsorbed water, H-O-H;				
	1646, 1608	1640	1645, 1607	***C-C vibration***: stretching vibration of aromatic rings; C=O (carbonyls) and C=C conjugation in stretching mode; C=C bond of aromatic dihydroxphenyl ring.		1646, 1609	1641, 1607	1646
	1578, 1519	1579, 1515	1517			1576, 1485	1579, 1515	
1451	1448, 1442	1449	1451	Distribution of C=C stretching bond of aromatic ring; asymmetric CH deformation vibrations.	1451	1449	1460	1463, 1451
1416	1423	1418	1414	C-O-H rocking stretching vibration or C-C-H, H-C-H and C-O-H modes of deformation; complex CH_2_OH-type vibrations.	1412	1413	1404	1412, 1407
1366	1375, 1357	1367	1366		1390	1398, 1371		1390
1335	1329	1337	1336	C-O-H bending vibration of heterocyclic bond or C-C stretching.	1332	1334	1330	1349
1299	1298	1299	1300	C-O-H bending vibration band; C-O-C of chromen structure.		1298	1301	
	1274	1276				1274	1275	
1246	1254		1248		1253	1255	1256	1249
***Vibrational band frequency region (1200–1000 cm^−1^)***								
1202	1199	1200	1203	Stretching vibration of C-O-H phenolic components.	1211	1205, 1197, 1174	1204	1205
1157	1143	1155	1157	C-O-C asymmetric stretching vibration coupled with bending C-O-H vibration mode.		1158, 1145		
	1116				1127	1123	1129, 1120, 1115	1125, 1109
1081	1078, 1044	1080	1080	C-O-H stretching mode (alcoholic).	1083	1078, 1068		1084
1030	1026	1031	1030	Overtones of C-H band with C-O-H stretching.	1046	1045		
1000		1000	1000		1010	1028		
***Vibrational band frequency region (1000–700 cm^−1^)***								
938	977	946	947	***Carbohydrates isolated components:*** Skeletal vibration involving linkage to fingerprint region.	946	997, 976, 959	995, 949	947
	889	885			926	929	927	928
858	871	861	860	Wagging-type vibration modes of hydroxyl bonds directly linked at sugar rings.	865	866	865	
	848				853		851	850
	815, 803					803		
758	762	758	760	Fingerprint region vibration of the C=O (carbonyls), and C-C bonds of the β-CD and glucopyranosyl ring structure.	776	760	764	776
	746				754	734		762
709	711	710	708		708	696, 672		708
					651	655, 624		645
					571	594, 542		560
					475, 440	499, 452, 413	480	498, 477; 439
					361	353	375,351	357
					320	321, 299		319, 310
						255, 215	252	256

**Table 2:** ^1^H-NMR chemical shift (δ) and peak descriptions of β–CD, the HEPT7G+βCD physical mixture, and the HEPT7G/βCD inclusion complex, and observed changes in chemical shift (δ) corresponding to β–CD protons in the HEPT7G+βCD physical mixture and the HEPT7G/βCD inclusion complex.

^1^H NMR Description		Chemical Shifts, ppm					
β–*Cyclodextrin Protons*	*Peaks*	β–*CD*	*HEPT7G+*β*CD* *Physical Mixture (PM)*	Δδ = δ_CD_ − δ_PM_ (ppm)	*HEPT7G/*β*CD Inclusion complex (IC)*	Δδ = δ_IC_−δ_PM_ (ppm)	Δδ = δ_IC_−δ_CD_ (ppm)
H1	[d]	4.954 (J_H_ = 3.8)	4.962 (J_H_ = 3.8)	0.008	4.934 (J_H_ = 3.8)	−0.028	−0.01
H2	[dd]	3.533 (J_H_ = 10.0)	3.535 (J_H_ = 10.0)	0.002	3.518 (J_H_ = 10.0)	−0.017	−0.015
H3	[t]	3.848 (J_H_ = 9.6)	3.851 (J_H_ = 9.5)	0.003	3.794 (J_H_ = 9.6)	−0.057	−0.054
H4	[t]	3.467 (J_H_ = 9.6)	3.47 (J_H_ = 9.4)	0.003	3.463 (J_H_ = 9.5)	−0.007	−0.004
H5	[dt]	3.747 (J_H_ = 8.6)	3.745 (J_H_ = 8.3)	−0.002	3.619 (J_H_ = 8.5)	−0.126	−0.128
H6	[dd]	3.778 (J_H_ = 13.9)	3.775 (J_H_ = 12.7)	−0.003	3.75 (J_H_ = 12.3)	−0.025	−0.028

**Table 3:** ^1^H-NMR chemical shift (δ) and peak descriptions of the HEPT7G+βCD physical mixture, HEPT7G/βCD inclusion complex, pure HEPT7G (standard), and hesperidin (raw material) along with ^13^C-NMR chemical shift data of the HEPT7G/βCD inclusion complex. The observed changes in chemical shift (δ) corresponding to protons of HEPT7G in the HEPT7G+βCD physical mixture and HEPT7G/βCD inclusion complex are also reported.

^1^H NMR Description		Chemical Shifts, ppm					^13^C NMR Description		Chemical Shifts, ppm
*Flavanone ring Protons*	*Peaks*	*HEPT7G/*β*C* *Inclusion Complex (IC)*	*HEPT7G+*β*CD Physical Mixture (PM)*	Δδ = δ_IC_−δ_PM_ (ppm)	*HEPT7G* *Standard*	*Hesperidin*	*Flavanone ring Carbons*	*Peaks*	*HEPT7G/*β*CD* *Inclusion Complex (IC)*
H-8 (–2S)	[d]	6.299 (J_H_ = 2.4)	6.307(J_H_ = 2.6)	−0.008	6.177	6.286	C-2	[d], [s]	69.606 and 79.427
H-8 (–2R)	[d]	6.229 (J_H_ = 2.3)	6.244(J_H_ = 2.6)	−0.015	nd	nd	C-3	[d], [d]	43.302 and 70.492
H-6 (–2S)	[d]	6.253 (J_H_ = 2.1)	6.267(J_H_ = 2.0)	−0.014	6.177	6.275	C-4	[d], [s]	73.178 and 197.99
H-6 (–2R)	[d]	6.213 (J_H_ = 2.0)	6.227(J_H_ = 2.0)	−0.014	nd	nd	C-5	[d], [s]	68.878 and 164.256
H-3	[dd]	2.919 ((J_H_ = 17.3)	Very Small	nd	2.810	2.921	C-6	[d], [d]	18.082 and 97.429
H-3 (auxiliary) (–2S)	[m]/[dd]	3.106 (J_H_ = 17.1)	3.099 (J_H_ = 17.5)	0.007	nd	nd	C-7	[s]	166.287
H-3 (auxiliary) (–2R)	[m]/[dd]	2.985 (J_H_ = 19.2)	3.017 (J_H_ = 17.2)	−0.032	nd	nd	C-8	[d]	95.060
H-2 (–2S)	[dd]	5.436 (J_H_ = 7.9)	5.452(J_H_ = 8.4)	−0.016	5.448	5.551	C-9	[s]	163.245
H-2 (–2R)	[dd]	5.283 (J_H_ = 10.2)	Very Small	nd	nd	nd	C-10	[s]	105.080
H-2′	[d]	6.785 (J_H_ = 4.4)	6.812 (J_H_ = 3.8)	−0.027	6.690	7.108	C-1′	[s]	132.526
H-3′	[dd]	6.755(J_H_ = 8.3)	6.779 (J_H_ = 7.9)	−0.024	6.690	7.094	C-2′	[d]	114.460
H-4′ (O-CH_3_ group)	[s]	3.696	3.756	−0.060	3.789	3.905	C-3′	[d], [d]	75.373 and 147.469
H-5′	[d]	6.729 (J_H_ = 2.0)	6.754 (J_H_ = 1.3)	−0.025	6.690	nd	C-4′	[s], [s]	57.475 and 149.263
H-6′	[d]	6.812 (J_H_ = 8.2)	6.839 (J_H_ = 7.8)	−0.027	6.690	7.094	C-5′	[d]	112.632
							C-6′	[d]	118.897
* Flavanone glucose protons:						**10 protons:**	***Flavanone glucose carbons:***		
H-1″	[t]	5.089 to 5.109; [5.009]	4.908 to 5.119; [5.014]	0.085	5.077 to 5.090	5.203 to 5.216	C-1″	[d]	98.745
H-2″	[m]	3.221 to 3.252; [3.237]	3.202 to 3.232; [3.217]	0.020	3.186 to 3.241	3.294 to 3.358	C-2″	[d]	72.493
H-3″	[m]	3.545 to 3.595; [3.570]	3.584 to 3.620; [3.602]	−0.032	3.491 to 3.549	3.580 to 3.635	C-3″	[d]	75.373
H-4″	[m]	3.259 to 3.312; [3.286]	3.341 to 3.372; [3.357]	−0.071	3.378 to 3.410	3.380 to 3.413	C-4″	[d]	70.957
H-5″	[m]	3.325 to 3.423; [3.374]	3.391 to 3.430; [3.411]	−0.037	3.450 to 3.505	3.511 to 3.541	C-5″	[d]	76.875
H-6″	[m]	3.647 to 3.678; [3.663]	3.633 to 3.681; [3.657]	0.006	3.629 to 3.658	3.695 to 3.736	C-6″	[t]	65.200

**Notes:** nd: not detected; [s]: singlet; [d]: doublet; [t]: triplet; [m]: multiplet; proton coupling constants are (J_H_ in Hz) in parenthesis. * [value] represents the average chemical shift (δ) of protons.

**Table 4:** ^13^C-NMR chemical shift (δ) of β–CD and the HEPT7G/βCD inclusion complex, and observed changes in chemical shift (Δδ) corresponding to β–CD carbons in the HEPT7G/βCD inclusion complex.

^13^C NMR Description	Chemical Shifts, ppm		
β–*Cyclodextrin* *Carbons*	β–*CD*	*HEPT7G/*β*CD* *Inclusion complex (IC)*	Δδ = δ_IC_−δ_CD_ (ppm)
C1	103.141	103.225	0.084
C2	73.353	73.361	0.008
C3	74.357	74.511	0.154
C4	82.412	82.266	− 0.146
C5	73.101	73.085	− 0.016
C6	61.560	61.322	− 0.238

**Table 5:** X-ray diffraction data of β-CD, the HEPT7G+βCD physical mixture, and the HEPT7G/βCD inclusion complex.

β-Cyclodextrin			HEPT7G+βCD Physical Mixture (PM)			HEPT7G/βCD Inclusion Complex (IC)		
2θ (Degree)	d-Value (nm)	Rel. Intensity (I/I_0_)	2θ (Degrees)	d-Value (nm)	Rel. Intensity (I/I_0,_ %)	2θ (Degrees)	d-Value (nm)	Rel. Intensity (I/I_0_, %)
6.233	1.41684	6.7	6.143	1.43753	10.9	12.685	0.69727	30.2
9.017	0.97994	62.7	8.924	0.99006	33.4	22.629	0.39262	50.6
10.682	0.82754	45.7	10.578	0.83563	36.7	23.577	0.37703	24.6
12.503	0.70735	100.0	12.396	0.71344	100.0	25.478	0.34932	22.5
14.667	0.60344	18.5	14.144	0.62563	63.4	31.796	0.2812	100.0
15.292	0.57895	13.8	15.284	0.57924	6.4	33.996	0.26349	50.3
15.415	0.57435	39.2	17.033	0.52014	42.0	37.838	0.23757	18.6
17.104	0.51799	46.3	18.686	0.47448	20.4			
17.653	0.502	13.9	18.848	0.47043	12.1			
18.925	0.46853	21.2	19.512	0.45457	29.0			
19.586	0.45287	30.8	21.052	0.42165	20.8			
21.205	0.41864	24.8	22.842	0.38899	53.8			
22.702	0.39136	42.2	24.187	0.36767	19.1			
24.266	0.36649	20.0	24.769	0.35915	4.9			
25.657	0.34691	23.7	25.426	0.35002	11.4			
27.102	0.32874	45.3	26.961	0.33044	30.6			
28.543	0.31246	12.3	28.629	0.31154	6.2			
30.985	0.28838	4.7	31.89	0.28039	11.9			
31.946	0.27992	15.7	34.608	0.25897	9.2			
34.74	0.25802	12.1	36.915	0.24329	4.5			
35.864	0.25018	10.2						
36.923	0.24325	4.9						

## Data Availability

Not available.
